# Towards quality-assured measurements of microplastics in soil using fluorescence microscopy

**DOI:** 10.1007/s00216-025-05810-6

**Published:** 2025-03-10

**Authors:** Quynh Nhu Phan Le, Crispin Halsall, Stoyana Peneva, Olivia Wrigley, Melanie Braun, Wulf Amelung, Lorna Ashton, Ben W. J. Surridge, John Quinton

**Affiliations:** 1 Lancaster Environment Centre, Lancaster University, Lancaster, LA1 4YQ UK; 2 Wessling GmbH, Am Umweltpark 1, 44793 Bochum, Germany; 3 Institute of Crop Science and Resource Conservation, University of Bonn, 53115 Bonn, Germany; 4 Department of Chemistry, Lancaster University, Lancaster, LA1 4YB UK

**Keywords:** Polymers, Soil organic matter, Fluorescence microscopy, Nile red staining

## Abstract

Fluorescence microscopy is increasingly seen as a fast, user-friendly, and high-throughput method for detecting microplastics (MPs) in soil; however, its effectiveness across diverse MP types and soil properties remains underexplored. This study tested a fluorescence microscopy–Nile red (NR) staining approach on eight MP types, covering both biodegradable and non-biodegradable plastics, in three size ranges (≤ 150 µm, 100–250 µm, 500–1000 µm) across loamy, clayey, and sandy soils. Each sample, processed in triplicate, underwent a relatively quick and straightforward extraction procedure involving density separation, organic digestion, and NR staining, followed by fluorescence and bright-field microscopy. A new digital image analysis pipeline using Image J was developed to expedite and (semi)automate MP quantification. Recoveries ranged from 80% to 90% for MPs with a Feret diameter of 500–1000 µm, regardless of soil type. In contrast, the recovery of smaller MPs (Feret dia. ≤ 250 µm) varied depending on the soils and plastic types: recoveries for low-density polyethylene (LDPE) reached 85% in sandy soil and 90% in loamy soil, whereas those for biodegradable polybutylene adipate terephthalate/polylactic acid (PBAT/PLA) were only 60% and 10%, respectively. The lowest recovery rate was observed in clayey soil and for biodegradable plastics. The method was tested on non-agricultural soil samples, yielding a MP mean number concentration of 20.7 ± 9.0 MPs/g for MPs sized from dia. ≥ 25 µm, comparable to Fourier transform infrared (FPA-µ-FTIR) results of 13.1 ± 7.3 MPs/g (*p* > 0.05). We conclude that fluorescence microscopy with NR staining and automated particle quantification offers a time-efficient, reproducible, and accurate method for MP detection in light-textured soils, whereas limitations remain for reliable MP analysis in clay-dominated soils.

**Graphical Abstract:**

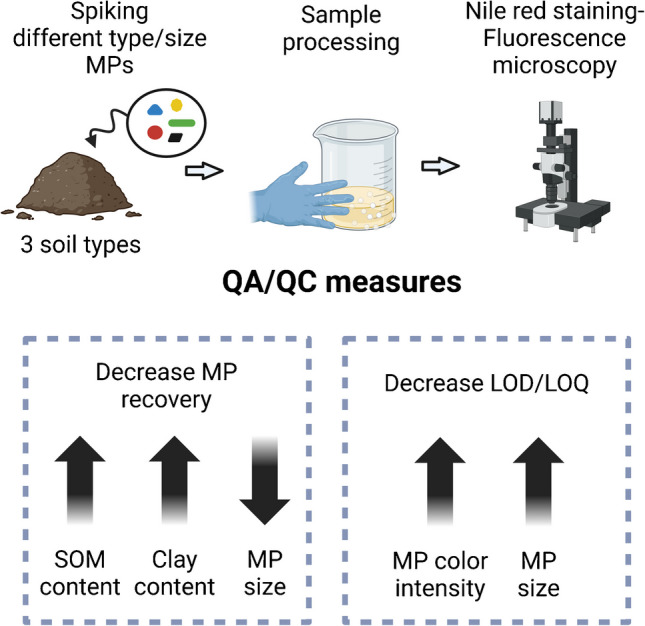

**Supplementary Information:**

The online version contains supplementary material available at 10.1007/s00216-025-05810-6.

## Introduction

Plastic debris is of increasing concern due to enormous levels of plastic production coupled with inefficient recovery and recycling of used plastic products. Plastic pollution has emerged as a major global environmental challenge in recent decades [1, 2]. In the environment, plastic debris disintegrates into smaller pieces through chemical, physiochemical, and biological processes. Those with dimensions ranging from 1 µm to 5 mm are defined as microplastics (MPs), accumulating readily in terrestrial and aquatic ecosystems, including freshwater, sediments, and soils, as well as within the atmosphere and in foodstuffs [3, 4]. While MP pollution in aquatic ecosystems has been widely studied, less research has focused on understanding the occurrence and fate of MPs in terrestrial ecosystems, particularly agricultural soils where plastics are frequently used (e.g., as mulching films or inadvertently applied through biosolid amendments) [5–7]. Reliable methods for detecting, characterising, and quantifying MPs in soil are essential for understanding their environmental fate and ecological impacts.

Fluorescence microscopy using Nile red (NR) staining has emerged as a low-cost, relatively simple-to-use approach for analysing a broad spectrum of MPs in complex environmental matrices like soils [8–11]. The fluorescent tag NR (9-(diethylamino)−5H-benzo[a]phenoxazin-5-one) is the most commonly applied fluorescent dye in MP research due to its strong fluorescence in a hydrophobic environment [12, 13]. However, the presence of lipophilic natural organic debris in soils can lead to false positives [11, 14, 15], which is an example of a potential source of error that needs to be examined further in tests soils with varying natural organic matter (OM) content.

The effectiveness of fluorescence microscopy–NR staining for detecting MPs is strongly influenced by the physiochemical properties of the plastics, including the presence of additives, which can vary widely, particularly in commercial plastics [9]. However, most research has concentrated on pristine MPs [11, 16, 17] resulting in a limited understanding of NR fluorescence in MPs derived from commercial sources. Moreover, larger-sized MP fragments (> 300 µm) are frequently utilised for method assessment due to their availability and ease of identification [18]. However, these larger MPs constitute only a minor proportion of the MPs present in the environment and are less relevant to ecotoxicological studies [18]. The behaviour of smaller MPs, especially in terms of their interaction with soil matrices, can affect the efficiency of MP extraction and identification from soil, yet our understanding of these processes remains limited.

Given the increasing interest in using fluorescence microscopy for measuring MPs in complex environmental matrices, this study aimed to establish an accurate, reproducible fluorescent-based methodology for analysing MPs in soil, which discriminates plastic size and type across different soil types. Here, a comprehensive appraisal of the method—based on spiking three different soil types with a variety of plastics of varying size categories (dia. ≤ 150 µm, 100–250 µm, and 500–1000 µm)—was undertaken. The method was then applied to non-spiked background soil samples (rural, non-agricultural soil) and compared to an infrared spectroscopy-based methodology. Our hypothesis is that a fluorescent microscopy–NR staining approach is suitable for analyzing MPs in soil, although the efficiency of this technique is influenced by polymer type and size, as well as by the soil characteristics.

## Materials and methods

### Preparation of soil and MP standards

#### Soil

Sandy and clayey soils (Cambisol and Stagnosol, respectively) were collected near Bonn, Germany, with sandy soil from an agricultural topsoil and clay soil from a forest subsoil [19], air-dried, and passed through a 2-mm sieve. Standard agricultural loamy soil (air-dried, 2-mm sieved, LUFA 2.4, LUFA Speyer, Germany) was also included for systematic method validation. The physiochemical properties of the LUFA soil were determined and provided according to good laboratory practices, whereas those for sand and clay soils were measured according to reference methods at Bonn University (Table S1). In addition, pasture-land soil (with no known recent agricultural amendments) was sampled from Lancaster University, Hazelrigg, meteorological field station and used as a non-spiked test soil.

#### Reference MP materials

A mixed MP standard was created for the recovery experiment, comprising various polymers across size ranges of dia. ≤ 150 µm (low-density polyethene, LDPE), dia. 100–250 µm (polybutylene adipate-co-terephthalate/polylactic acid blend (PBAT/PLA), and dia. 500–1000 µm. For small microplastics (SMPs, ≤ 250 µm), LDPE and PBAT/PLA were used to represent conventional and biodegradable MPs. LDPE particles (≤ 150 µm) were sourced from Goonvean Fibres Ltd, UK, while PBAT/PLA particles (100–250 µm) were cryo-milled and sieved from biodegradable mulching films (Bionov Black, Barbier Group, France).

The larger microplastics (LMPs, dia. 500–1000 µm) are selected from polymer materials that represent the most commonly encountered synthetic polymers in the environment, including low-density and high-density polyethene (LDPE and HDPE), polypropylene (PP), polystyrene (PS), polyethene terephthalate (PET), polyamide (PA), polyvinyl chloride (PVC) [13], and the biodegradable plastic PBAT/PLA. These LMPs were prepared using a razor blade from consumer items like milk bottle caps and food packaging, as listed in Table S2. They were distinguishable for separation from non-spiked MPs and external contaminants, with polymer types confirmed by attenuated total reflectance Fourier transformed infrared spectroscopy ATR FTIR (Lumos II, Bruker) and Raman micro-spectroscopy (WITec alpha 300R, Oxford) (Fig. S1).

Each 10 g of soil was spiked with MPs to achieve a MP concentration below 0.1% (w/w). Forty LMPs (five of each polymer type) were counted, photographed, and fixed to a gelatin plate (1 × 1 cm, Dr. Oetker Blatt Gelatine) using a needle and fine‐point high‐precision forceps. Further, 3 mg each of PBAT/PLA (dia. 100–250 µm) and LDPE (dia. ≤ 150 µm) particles were encapsulated in gelatin “dumplings” for loss-free soil incorporation (Fig. S2 and S3) [20]. The gelatin plate and “dumplings” were added to soil and shaken in water and zinc chloride (ZnCl_2_) solution for 2 h at room temperature to dissolve the gelatin and release the MPs for subsequent extraction.

### Extraction of MPs from soils

**Fig. 1:**
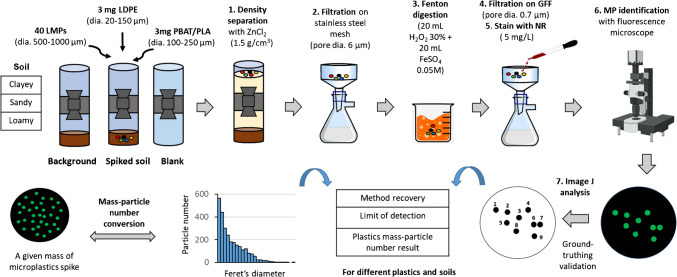
A schematic showing the extraction and separation steps of the MP-soil measurement procedure

MPs were extracted from the soil, starting with a density separation step (Fig. 1) using a 1.5 g cm^−3^ ZnCl_2_ solution (≥ 97%, APC Pure, UK) in a Sediment Microplastic Isolation (SMI) unit (Fig. S4) [21]. The pre-cleaned unit was filled with 10 g of soil and 50 mL of ZnCl_2_, shaken for 2 h, and settled overnight after an additional 200 mL of ZnCl_2_. The supernatant was filtered on a stainless-steel mesh (pore dia. 6 µm, 47-mm Ø, GKD industrial, Germany), rinsed with HPLC-grade water (H_2_O, Fisher Scientific, UK), and Zn precipitates dissolved with 10% sulfuric acid (H_2_SO_4_, Fisher Scientific, UK). The filter was placed in a beaker containing 0.05 M iron (II) sulphate heptahydrate solution (FeSO_4_.7H_2_O, ≥ 99%, Acros Organics, UK), sonicated, and rinsed with HPLC-grade water to collect the extracted particles from the filter. This was followed by the addition of 20 mL of 30% hydrogen peroxide (H_2_O_2_, Fisher Scientific, UK) to initiate a Fenton reaction [20, 21]. After 24 h, samples were filtered onto a glass fibre filter (GFF, pore dia. 0.7 µm, 47-mm Ø, Cytiva Whatman GF/F, Fisher Scientific, UK), stained with 5 mg L^−1^ NR (C_20_H_18_N_2_O_2_, 99%, Acros Organics, UK), rinsed with hexane (C_6_H_6_, ≥ 95%, Fisher Scientific, UK), and dried in the dark. Further details of this extraction procedure are provided in the supporting information.

### Fluorescence microscopy and image analysis

Fluorescence microscopy imaging was performed using a Zeiss Axio Zoom.V16 microscope equipped with a macro lens, 12 MP camera, and automated stage. In a darkroom, samples stained with NR were illuminated at 470 nm and observed through the green filter set (emission 524/50 nm). Images were captured in green fluorescence and BF modes at 50 × magnification using Zen Pro software’s stitching function and surface focusing.

Particle recognition and quantification were performed in Fiji-ImageJ 1.53t (https:// imagej. nih. gov/ ij/, accessed 21 October 2022), as described in Fig. S5. Images were imported in Carl Zeiss format (czi) and processed in Tag Image File (tif) format. A Gaussian blur filter was applied to smooth the image, followed by subtraction from the original to minimise noise and reduce detection runtime. Global thresholding segmented particles from the background, with specific grey values set for different experiments. A fill-hole operation compensated for particle penetration, and watershed segmentation split agglomerated fluorescent particles. For BF images of PBAT/PLA, watershed segmentation was avoided to prevent overcounting caused by irregular shapes. Particles were quantified and classified based on Feret’s diameter. LMPs (500–1000 µm) were manually counted due to their varying fluorescence, diverse shapes, and low abundance. Further details of the detection protocols are provided in the supporting information.

#### Evaluation of the image segmentation algorithm

All procedures performed in studies involving human participants were in accordance with the ethical standards of the institutional and/or national research committee and with the 1964 Helsinki Declaration and its later amendments or comparable ethical standards.

*Accuracy* or *validity* was assessed by comparing manual counts of particles by a human expert with algorithmic counts from Image J. For both fluorescence and BF images, the accuracy—indicating the correct identification rate of particles and background—and sensitivity—reflecting the true positive rate of particle detection—were determined using the following equations [22]:

1 $$\text{Accuracy }(\text{\%})=\frac{{N}_{\text{true possitive}}+{N}_{\text{true negative }}}{{N}_{\text{true positive}}+{N}_{\text{true negative}}+{N}_{\text{false positive}}+{N}_{\text{false negative}}}\times 100$$

2 $$\text{Sensitivity }\left(\text{Recall},\text{ \%}\right)= \frac{{N}_{\text{true possitive}}}{{N}_{\text{true positive}}+ {N}_{\text{false negative}}}\times 100$$

where *N*_true positive_ and *N*_false positive_ are the number of correctly or incorrectly identified particles, respectively, and *N*_true negative_ and *N*_false negative_ are the number of particles identified correctly or falsely as background pixels, respectively. *Precision* (*or reliability*), which is the ability of the algorithm to yield the same results from repeat scans of the same image, was assessed by rotating the filter image by 90° counterclockwise, vertically flipping the original image, and comparing the results to the initial scan.

### Quality assurance and quality control

#### Plastic mass versus particle number relationship

Small microplastic particles (SMPs) were spiked at a specific mass instead of particle number, due to the difficulty in handling particles with dia. ≤ 250 µm. SMP particle concentration was determined by introducing 5.0 mg of each reference material (LDPE and PBAT/PLA) to a 100-mL volumetric flask filled with ethanol (five replicates). Samples were sonicated for 15 min at room temperature to ensure particle dispersion. Next, 10 mL aliquots were vacuum-filtered onto GFFs. LDPE particles were subsequently stained with NR, while PBAT/PLA particles were not stained due to the absence of a detectable fluorescence signal in previous experiments (Fig. S6) and were instead analysed in BF mode. Both types of SMPs were examined under a fluorescence microscope, followed by ImageJ analysis as previously described, to assess their particle concentration relative to mass and size distribution.

Additionally, particle size distributions of SMPs in ethanol suspension were measured using a Syringe particle counter equipped with a laser diode sensor LDS 30/30 and SW-PE evaluation software (Markus Klotz GmbH, Germany). Each 1.5 mL suspension sample was mixed with a magnetic stirrer to ensure even particle distribution. Measurements were conducted on five replicates per sample, and results were reported as mean values.

#### Quality control

Strict quality control and assurance measures were implemented to ensure adherence to reporting guidelines for validity, reproducibility, and comparability, covering key components such as replicates, detection limits, blank controls, positive controls, relative standard deviation (%RSD), and contamination mitigation [23]. Non-plastic laboratory equipment was used, when possible, cleaned several times with HPLC gradient grade water and acetone (CH_3_)_2_CO, ≥ 99.5%, Acros Organics, UK), and covered with clean aluminum foil. All GFFs, stainless-steel meshes, and non-volumetric glassware were baked at 500 °C for 4 h before use. 100% cotton pink-dyed lab coats and nitrile gloves were worn during sample handling. Procedures were conducted in a fume hood thoroughly cleaned with HPLC water and acetone. All reagents, including ZnCl_2_, H_2_O_2_, and FeSO_4_ solutions, were filtered through GFFs before use. Routinely, laboratory blanks (triplicate) were processed with each batch of samples, undergoing the same procedures as the spiked and background samples in order to monitor potential contamination.

The limit of detection (LOD) was determined for different MP sizes as the mean of blanks (*n* = 3) plus three standard deviations (SD), and the limit of quantification (LOQ) was calculated as LOD + 10SD. To assess the method’s precision, triplicate spiked soil samples were analysed, and the relative standard deviation (%RSD) was calculated for each soil type.

### Validation of the fluorescent staining protocol on rural soil samples

Six soil samples were randomly taken from the Hazelrigg field station (54.01° N, 2.77° W) near to Lancaster University campus in northwest England. The site has been free from tillage farming practices for decades. A 4 m margin along the field borders was avoided to prevent contamination from adjacent areas or dirt roads. Each sample was collected from a depth of 0–20 cm within a 50 × 50 cm quadrat and thoroughly mixed with a stainless-steel shovel. Subsamples (1–2 kg) from each site were stored in 100% cotton bags and kept at 4 °C. Soil physicochemical properties are detailed in Table S3. For MP extraction, 50 g of each soil sample was dried at 40 °C and sieved through a 2-mm mesh. The sieved soil was then subjected to the described MP extraction process, samples analysed using fluorescence microscopy, as well as Fourier transform infrared (FTIR) microscopy coupled with a focal plane array (FPA) detector in the transmission mode. Detailed procedures for soil sampling and FPA-µ-FTIR analysis are provided in the supporting information.

## Results and discussion

### Recovery of various large microplastic particles (LMPs, dia. 500–1000 µm)

#### Fluorescence of test polymers stained with NR

The NR fluorescence strength varied for different LMPs under the green filter, with variations in intensity based on each polymer’s physiochemical properties (Fig. S6 and Fig. S7). Black PBAT/PLA from the mulching film did not fluoresce with NR and brown PA fragments from the fishing line showed a weak fluorescence signal before the extraction. This is consistent with the observations of Stanton et al. [10], where brown HDPE and black PP were stained only around their edges, whereas red PA, black polyester, and blue acrylic fibres were not stained by NR. The authors suggested that the presence of plastic dyes affected the affinity for NR.

Interestingly, our research did not find a direct link between polymer hydrophobicity and fluorescence intensity. This differs from previous reports suggesting that plastics with greater hydrophobicity, such as PE, PP, and PS, generally exhibit stronger fluorescence than less hydrophobic surfaces, like PET, PU, and PVC [16, 17, 24]. In our research, plasticised PVC from insulated cables displayed the most intense fluorescence, outperforming PS, LDPE, PET, PP, HDPE, and PA.

Understanding NR’s fluorescence mechanism is crucial for elucidating its varying behaviour in different polymers. Upon excitation, NR can adopt either the twisted intramolecular charge transfer (TICT) state or the planar intramolecular charge transfer (PICT) state, influenced by the rotation or electron movement leading to cross conjugation within its diethylamino group [25–29]. This results in distinct fluorescence behaviors due to molecular interactions with NR’s π electron system. Moreover, interactions such as π-π, electrostatic interactions, hydrogen bonding, van der Waals forces, and pore-filling affect NR’s sorption on MPs, which vary based on carrier solvents, polymer crystallinity, and functional groups [30].

The strong fluorescence of NR in PVC can be attributed to the polymer’s increased flexibility due to the presence of plasticisers, which reduce the intermolecular forces between polymer chains and increase chain mobility (free volume of polymer) [30]. This allows the plasticised PVC to absorb NR into its bulk polymer due to the potential for segmental chain movements and allows for TICT conformation of excited NR, as the diethylamino groups of NR have more freedom to rotate. Nel et al. [24] also found stronger fluorescence of NR in plasticised PVC compared with rigid PVC, although the reason for this difference was not discussed. Polystyrene also showed strong fluorescence of NR, not only due to its porous structure but also its aromatic groups allowing for π–π interactions with NR, leading to increased NR sorption capacity.

In contrast, highly rigid MPs such as HDPE and PET exhibit weaker fluorescence with NR due to limited chain flexibility, as NR absorbs more on the surface than in the bulk polymer. Heating polymers to their glass transition temperature (*T*_*g*_) enhances NR fluorescence by reducing cohesive forces and cross-linkages, thus increasing chain mobility and free volume for NR sorption [11, 31–34]. However, determining precise *T*_*g*_ values is complex due to factors like molecular weight, polar groups, side group immobility, chemical structure crosslinking, and the presence of moisture and plasticisers [30]. Heat treatment can also lead to physiochemical changes, such as PE blackening at 100 °C [30]. In our study, staining was performed at room temperature to avoid potential adverse effects on PBAT/PLA. Additionally, treatments like Fenton’s reagent and possibly acidic ZnCl_2_ might increase NR absorption capacity, likely through minor surface modifications, additive leaching, etc. (see “Recoveries of LMPs (dia. 500–1000 µm) from soils”).

Indeed, caution is crucial when classifying and identifying MPs based solely on their NR fluorescence intensity. This is because NR fluorescence in MPs is affected by various factors, including the chemical characteristics of plastics, such as polarity, crystallinity, functional groups, presence of additives, and staining conditions like NR concentration, carrying solvents, and heating conditions (when applied). This complexity also underscores the need for careful evaluation, especially when considering weathered or contaminated MPs.

NR fluorescence variability among plastics also complicates MP segmentation followed by (semi)-automatic quantification using digital color data. Setting appropriate pixel brightness thresholds is challenging, as it must distinguish polymer fluorescence from interfering substances like organic residues while ensuring accuracy and reproducibility. In our study, HDPE fragments showed lower fluorescence, and PP and LDPE films fluoresced strongly only at the edges, making threshold settings difficult. Erni-Cassola et al. [17] found that lower thresholds increased the recovery of polymers like PS, PE, PP, and Nylon-6 but also caused false positives from natural OMs like wood and chitin, and errors from halo effects and particle merging. Nel et al. [24] recommended a minimum brightness threshold of 100 arbitrary units to include polymers like expanded polystyrene (EPS), HDPE, PP, and Nylon-6 while excluding natural fluorescing matter. However, this threshold excludes highly crystalline plastics like PVC and PET and may miss dimly fluorescing plastics and small particles. Longer exposure times for enhanced fluorescence can also lead to oversaturation and data loss [30]. Therefore, manual counting was applied for LMP fractions to ensure accurate method validation and circumvent digital analysis errors for a wide range of plastic types. However, future advancements in machine learning and artificial intelligence might offer potential solutions for these issues [30].

#### Recoveries of LMPs (dia. 500–1000 µm) from soils

**Fig. 2:**
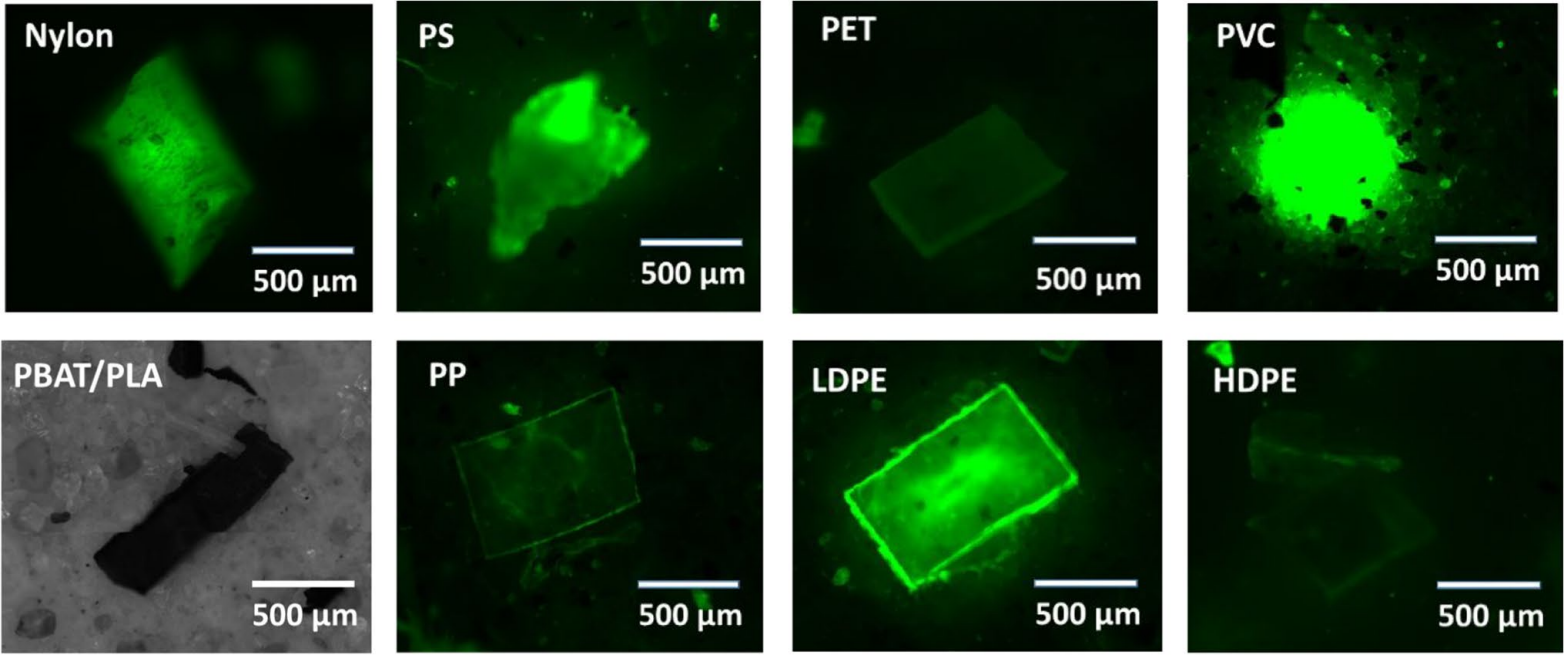
Fluorescence image of different polymer types (from left to right: nylon, PS, PET, PVC, PBAT/PLA, PP, LDPE, HDPE) with dia. 500–1000 µm after being extracted from the soil matrix. The MPs were stained with NR, illuminated under 470 nm, and observed through a green filter set. Black PBAT/PLA, which exhibited no fluorescence signal, was captured and analysed in brightfield mode

Approximately 90% of the LMPs were successfully recovered across all soil types (Figs. 2 and 4), demonstrating the effectiveness of the MP extraction method coupled with NR staining for most polymers, except for PBAT/PLA and highly rigid HDPE. The use of a ZnCl_2_ solution with a density of 1.5 g.cm^3^ was sufficient for extracting most plastic types tested within this size range. Our results also indicate negligible interference from soil-MP interactions for MPs in this size category under the tested conditions. However, this observation is specific to the interaction duration and conditions used in this research. Longer MP-soil interaction periods or multiple wet-dry cycles, which enhance soil-MP contact, could lead to different outcomes in terms of extraction efficiency and NR detection.

**Fig. 3:**
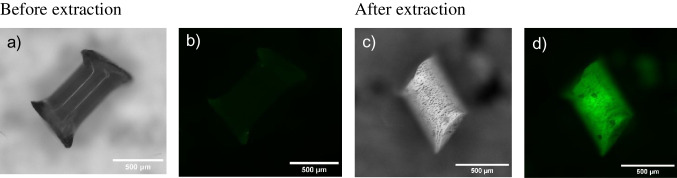
Bright-field (**a**, **c**) and fluorescence images (**b**, **d**) of nylon before and after extraction from soil, respectively

After extraction from the soil matrices, nylon fibres from the fishing line showed noticeable surface damage characterised by hole formation and increased fluorescence intensity (Fig. 3), phenomena not distinctly observed for other spiked polymer types. This enhanced fluorescence could result from changes in the polymer structure, such as splitting of the polymer backbone at the C-N linkages leading to alterations in the chemical structure of the polymer, changes in molecular orientation or local order, and/or from the loss of associated materials or additives [35]. PA degradation might be caused by acidic ZnCl_2_ and/or the oxidative Fenton reaction. Although previous studies have not observed PA degradation due to ZnCl_2_, some research has noted fragmentation and degradation of PA under specific acidic and alkaline conditions [29, 30].

Importantly, our research emphasises the sensitivity of NR toward changes in polymer hydrophobicity, rigidity, free volume, associated materials, etc., which could be utilised for plastic degradation in future studies.

Previous studies reported the shrinking of PE, PET, and biodegradable polymers, particularly PLA-based MPs, upon Fenton digestion, similar to the effects of H_2_O_2_ [36–38]. However, in our research, no noticeable changes were observed for LMPs except for LDPE, which had enhanced fluorescence of NR after extraction. Indeed, the Fenton reagent has been reported to remove natural OM efficiently and to have a milder impact on MPs than other treatments, such as alkaline and acidic methods [36, 37, 39]. While combining oxidative and enzymatic digestion is considered the most effective natural OM removal method, it is expensive, time-consuming, and can degrade some biodegradable MPs [38, 40].

### Recovery of small microplastics (SMPs, dia. 20–250 µm)

#### Particle concentration of spiked material

For LDPE (dia. 20–150 µm), the particle concentration determined by the microscopic method was 4900 ± 1100 particles/mg, while for the PBAT/PLA (dia. 100–250 µm), it was found to be 2300 ± 140 particles/mg. These particle numbers were utilised to establish SMP recoveries from different soil types. To this end, introducing 3 mg of LDPE dia. 20–150 µm or PBAT/PLA dia. 100–250 µm into the soils corresponds to approximately 14,700 ± 4500 particles and 6900 ± 420 particles, respectively (Fig. S8).

Notably, the results from the image analysis of fluorescence microscopy align well with those from the particle counter in the liquid phase, demonstrating the effectiveness of NR staining for small LDPE particles followed by subsequent image analysis. The average particle numbers found using the particle counter were 15,730 ± 140 particles in 3 mg for LDPE in a size range of 20–150 µm, and for PBAT/PLA, 3200 ± 30 particles in 3 mg in the size range of 100–250 µm (Fig. S9). For PBAT/PLA, a higher particle count was observed with the microscopic method compared to the particle counter. This may be due to difficulties in particle segmentation caused by irregular film fragment shapes and background contrast. Additionally, the discrepancy between the two methods could be influenced by substantial variations in PBAT/PLA particle size, as the particles become more fragile after cryo-milling.

The size distribution of the spiked LDPE (dia. ≤ 150 µm) and PBAT/PLA (dia. 100–250 µm) showed inconsistencies between the theoretical sieve mesh size and the actual particle sizes on the filter, with most particles being smaller than expected, regardless of plastic type (Fig. S8 and Fig. S9). This is possibly due to the spontaneous self-assembly of SMPs into crystal superstructures upon solvent evaporation [41, 42], a phenomenon that becomes less frequent when the polymer powder is diluted and sonicated during size characterisation. These very small particles contribute negligibly to the MP mass but are significant in contributing to the overall particle number for any given plastic type [43]. In the research reported here, for consistency, we only considered the sieved size ranges when assessing the recovery of SMPs.

Validation of the microscopic method combined with Image J analysis for automated particle segmentation and quantification of SMPs was established for comprehensive quality assurance. A comparison with manual counting for a section of the whole filter of only spiked SMP gave an agreement of 88% for LDPE and 78% for PBAT/PLA. Reliability or precision, based on repeated scans of the same image but at different angles (see Sect. "Quality assurance and quality control" above), revealed no differences in particle count. Under 50 × magnification, the smallest detected particle for spiked fluorescent LDPE and black PBAT/PLA particles (in BF mode) had a Feret’s diameter of 3.5 µm. However, a 20 µm size threshold was set for assessment of the method recovery of LDPE particles (dia. ≤ 150 µm) due to large uncertainty in measuring smaller fluorescence particles (Fig. S8) and increased soil matrix interference below this threshold (Fig. S13).

This study presents an image analysis pipeline designed for the reliable quantification of fluorescent particles in complex backgrounds. While fully automated approaches, such as MP-VAT [44, 45], provide quick and standardized solutions, the method proposed here offers a flexible framework that can be adapted to different sample characteristics. Depending on the nature of the sample, specific steps can be included or omitted to enhance accuracy. For example, compared to MP-VAT [44, 45], which primarily relies on colour thresholding for particle detection, this study incorporates additional steps to address potential background interference. Such interference may arise from incomplete dye washing, filter artifacts, or the halo effect from highly fluorescent particles, which can obscure adjacent particles. To mitigate these effects, Gaussian blur was applied, followed by thresholding and a fill-hole function to minimize particle loss during segmentation—particularly in cases where fluorescence occurs only at the particle edges, which is especially relevant for certain plastic films (Fig. 2), leading to the loss of the core. Additionally, watershed segmentation was selectively implemented to improve the identification of clumped particles. By integrating these steps, the proposed approach aims to reduce false detections caused by background artifacts and enhance the identification of clumped particles, providing a complementary method for improving the accuracy of fluorescence-based particle analysis (see Fig. S10).

#### Recovery of SMPs from different soil types

The recovery of SMPs was determined by calculating the number of particles in spiked soil samples (both fluorescence and BF modes) after subtracting the number of particles found in non-spiked (background) soil, expressed as a percentage of the initial number of spiked SMP particles in 3 mg (Fig. S11). The number of fluorescence particles (dia. 20–150 µm) found in background soils after blank correction was 880 ± 800, with clayey soils having the highest count, followed by loamy (LUFA 2.4) and sandy soils (Table S4). These fluorescence particles might represent the “native MPs” but could also be the residual natural OM particles being co-stained, as they were positively correlated with the soil OM content (*R*^2^ = 0.998, Fig. S12). The size distribution of fluorescent particles in the background soils (clayey, sandy, and loamy soils) shows an increasing number of MPs in the size range of ~ 20–60 µm (Fig. S13).

The number of black particles detected in background soils was relatively low, totaling 26 ± 30, with clayey soil having the highest count, followed by loamy and sandy soil. These black particles could be plastics (e.g., tire rubber) or black carbon (i.e., soot and char formed during incomplete combustion of fossil and biomass fuels). The fluorescence image shows that these black materials were not labelled with NR, thus interfering only with the detection of black PBAT/PLA in the BF mode.

Although Fenton treatment was applied, incomplete natural OM removal led to a high level of fluorescent particles in the background soil, potentially causing false positives if no background subtraction had been applied. This underscores the need for improvement of natural OM removal, possibly by employing more effective oxidative digestion, multiple Fenton treatments, or the addition of enzymatic treatments. However, caution is necessary as more aggressive removal of natural OM can adversely affect the MP particles. Advanced image analysis using machine learning has shown promise in distinguishing plastics from natural OM [46–48]. Additionally, incorporating hydrophilic water-based dyes such as Methylene Blue, Calcofluor White, Evans Blue, and DAPI alongside NR can help distinguish natural OM from NR-stained MPs, improving differentiation [15, 34, 49]. Ongoing research and the refinement of color thresholding techniques also hold promise for more effectively distinguishing MPs from natural OM [30].

**Fig. 4:**
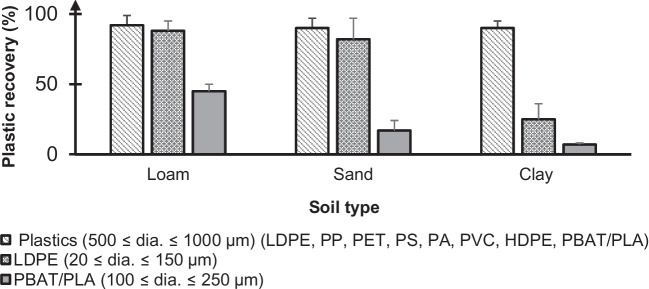
MP recovery for different polymers and MP size classes across the three soil types. Percent recoveries are reported as a mean of three replicates with standard deviation error bars

The recovery rates for SMPs are strongly dependent on soil type (Fig. 4), polymer type, and size range. LDPE (dia. 20–150 µm) achieved a relatively high recovery rate of approximately 82 ± 15% in sandy soil and 88 ± 7% in loamy soil (standard LUFA 2.4). In contrast, PBAT/PLA (dia.100–250 µm) had lower recovery rates for all soil types, measuring 17 ± 7% in sandy soil and 45 ± 20% in loamy soil. For both polymer types, the recovery of SMP particles was notably lower in clayey soil, with LDPE at 25 ± 11% and PBAT/PLA at 7 ± 1%.

The results show that compared to SMPs, LMPs were more efficiently extracted from soils, as evidenced by their higher recovery rates, which consistently exceeded 90%. Several factors contribute to the potential loss of SMPs during sample processing, such as particles sticking to glass beakers or glass filtration holders since these small particles are invisible to the naked eye. Additionally, SMPs may adhere to the surface of LMPs or plant debris after being concentrated on a filter, making their detection and quantification challenging through image analysis. These potential issues could be addressed by applying finer soil sieving before processing and by improving the organic digestion step. Furthermore, it is highly recommended to use lower MP concentrations per filter, either by subsampling or using larger filter diameters, to prevent particle agglomeration.

The recovery of SMPs also appears to be influenced by MP type. For instance, while small LDPE (dia. 20–150 µm) exhibited relatively high recovery rates in sand and loamy soil, lower recoveries were observed for the biodegradable PBAT/PLA MPs. This difference can likely be attributed to the increased degradation of biodegradable plastics relative to non-biodegradable plastics during Fenton’s digestion step, and during the subsequent ultrasonication after filtration on the stainless steel meshes which could lead to mechanical degradation and the loss of PBAT/PLA into smaller fractions. Notably, PBAT/PLA MPs were cryo-milled from thin mulching films and wet-sieved with acetone and thus were easily fragmented to much smaller sizes.

Soil characteristics play a crucial role in the extraction and quantification of SMPs. Among all soil types, clayey soil was associated with the lowest recovery, likely due to its higher natural OM (5.9%) and clay content (52%). Incompletely digested natural OM, particularly large plant debris, can obscure SMPs on filters, hindering microscopic detection and leading to lower recovery rates [50]. Furthermore, clay minerals consist of silica and aluminium layers. These are often negatively charged, either due to isomorphic replacement within the mineral structure or from the dissociation and protonation of chemical groups along their edges [51]. This characteristic results in the hetero aggregation between MPs and clay particles, as previously observed [52]. The hetero-aggregation process of MPs, influenced by factors like hydrochemical conditions and mineral types, is primarily driven by electrostatic interactions. High cation concentrations, particularly divalent ones (Ca^2+^, Mg^2+^, Zn^2+^), promote MP aggregation by neutralising their negative charge, as per DLVO theory [52–54]. Therefore, we propose that using ZnCl_2_ as a density separation medium could enhance MP-clay aggregation, especially in clay-rich soils, raising concerns about potential MP loss. However, further studies are needed to explore the specific impacts of ZnCl_2_ on MP-clay mineral aggregation in such soils, as it is currently one of the most used salts in MP separation [21, 55–58].

The relative standard deviation (%RSD), a method repeatability (precision) measure, varied with soil types and MP characteristics. For small LDPE (dia. 20–150 µm), %RSDs were 5% in loamy, 18% in sandy, and 60% in clayey soil. Biodegradable PBAT/PLA showed higher %RSDs: 10% in loam LUFA 2.4, 50% in sandy, and 60% in clayey soil. These values are substantially higher than those in conventional chemical testing and for LMPs (dia. 500–1000 µm), which range from 5 to 10% depending on soil type. This is attributed to soil sample heterogeneity, which affects the level of interference associated with factors such as natural OM and clay content. Uniformly spiking and effectively separating MPs from complex matrices is also challenging. In experiment “Particle concentration of spiked material”, inconsistencies were observed in delivering a uniform number of particles (%RSD for LDPE and PBAT/PLA was 25% and 10%, respectively) when pipetting from a stock suspension. Image analysis presents additional difficulties, particularly with smaller particles. Watershed segmentation helps resolve particle clumps but can also lead to the over-segmentation of LMPs, especially co-stained plant material and natural fibres with uneven fluorescence intensity. Therefore, obtaining a robust automatic image analysis becomes very challenging when a wide range of plastic sizes and types occur in organic-rich matrices.

The blanks for the entire protocol revealed an average of 130 fluorescence particles (dia. ≥ 20 µm) and none for black particles (dia. 100–250 µm), suggesting possible contamination of MPs from PVC density separation kits or airborne sources during processing. Notably, the number of particles in the blank is substantially lower than in the background and spiked soil samples. The method LOD varied with the MPs’ cut size and color intensity. For instance, the LODs for small LDPE particles were 570, 190, 130, and 50, and the LOQs were 1000, 330, 220, and 80 for cut sizes of 20 µm, 40 µm, 50 µm, and 100 µm, respectively. The LOD for PBAT/PLA with a 100 µm cut size was much lower than for small transparent LDPE particles, likely due to the enhanced detectability of its black color during sample processing, which warranted greater caution.

The selectivity of fluorescence techniques for different polymer types is clearly limited, unlike spectroscopic methods such as FTIR and Raman, which can determine polymer types with reasonable accuracy for individual MP particles. The data we report here highlight that some plastics, such as PET and HDPE, have reduced selectivity because they exhibit low fluorescence intensity when stained with NR, making it challenging to distinguish them from other matrix interferences (e.g., co-stained natural OM). To assess method selectivity more precisely, other techniques for chemical identification of MP particles can be employed and compared with fluorescence microscopy results. While relatively few studies have conducted such comparisons between NR-fluorescence microscopy and FTIR/Raman spectroscopy, there is generally good agreement between the two methods [11, 59].

The complete microplastic (MP) analysis protocol described in this study took approximately 2 days per sample, excluding pre-processing steps such as soil sieving, drying, and MP spiking. A batch of six samples, including procedural blanks, can be processed simultaneously. This included 12 h for density separation, 24 h for Fenton digestion (for a batch of 6 samples), and about an hour for staining and capturing images for one sample filter. This is much faster than most existing procedures, e.g., where enzymatic digestion is applied [20, 60, 61]. The complexity of these procedures, however, is justified for better organic matter removal, particularly when using spectroscopy methods like FTIR and Raman, where higher particle count on a filter can significantly increase the analysis time.

### Comparison of fluorescence microscopy with FTIR spectroscopy

**Fig. 5:**
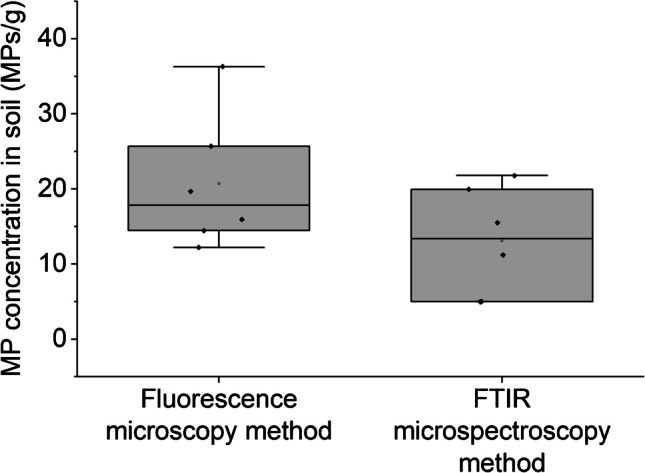
Box and whisker plot displaying the MP number concentrations in soil samples (*n* = 6) collected from the Hazelrigg field station, as measured by fluorescence microscopy and FTIR spectroscopy methods

The mean concentration of MPs detected in soil samples from the research station fields using NR staining with fluorescent microscopy was 20.7 ± 9.0 MPs/g. In comparison, the FPA-µ-FTIR method, after blank subtraction, showed a concentration of 13.1 ± 7.3 MPs/g (Fig. 5). A detailed comparison of each sample is provided in Fig. S14. Both methods detected MP levels above the limit of detection (LOD) in all samples. FTIR analysis identified several types of MPs, including polyethylene (8.6 MPs/g), polyester (0.31 MPs/g), polypropylene (1.25 MPs/g), polystyrene (0.80 MPs/g), styrene-butadiene rubber (3.1 MPs/g), polyvinyl chloride (2.4 MPs/g), and polyamide (0.1 MPs/g).

To the best of our knowledge, previous studies have not reported MP concentrations for non-agricultural soils with particles as small as 25 µm using fluorescence microscopy methods. However, the results of this study are consistent with those reported by Tagg et al., who found 6.39 MPs/g in a background (non-amended) soil from a research station in Germany using a similar FPA-µ-FTIR method, comparable to our FTIR approach [62].

Although the MP concentration detected by fluorescence microscopy was slightly higher than that by FTIR, a paired *t*-test showed no significant difference between the two methods (*p* = 0.153, two-tailed test). Slightly higher counts for the fluorescence method could be due to false positives arising from NR co-staining with soil organic matter. Similarly, FTIR spectroscopy may experience matrix interferences, leading to false positives from polymers like polyethylene, ethylene vinyl acetate, acrylics, and polyurethane [62–65]. Regarding true negatives, fluorescence microscopy struggles to detect black or dark-coloured MPs or those with physicochemical properties that weaken NR fluorescence as discussed above. FTIR can miss particles below the infrared diffraction limit or particles that are too thick, especially in transmission mode. FTIR also has challenges in detecting weathered MPs because weathering can alter their spectra, making them unrecognizable in spectral libraries. However, weathering increases surface sorption and decreases polymer crystallinity, which can enhance NR staining’s ability to detect these particles, as shown in our study.

## Conclusions

Fluorescence microscopy with NR staining and a semi-automatic particle recognition pipeline provides a reproducible and accurate method for counting MPs in heterogeneous matrices like soil. This method is suitable for routine soil analysis, accommodating a wide range of plastics (excluding black/dark-coloured plastics and less ideal for highly rigid plastics) with dia. ≥ 20 µm. Although the method has limitations in terms of polymer selectivity, enhancing the removal of natural OM can help reduce false positives. This study also underscores the impact of soil properties and MP characteristics, especially size and polymer type, on MP extraction efficiency. Biodegradable plastics (dia. 100–250 µm) had lower recovery rates from soil compared to non-biodegradable plastics (dia. 20–150 µm), possibly due to degradation during sample preparation. Soils with a higher OM content and very small and negatively charged mineral particles are complex matrices from which to extract MPs.

Additionally, the use of corrosive zinc chloride during density separation and the strong oxidative reaction of Fenton’s reagent may have adversely affected plastics susceptible to degradation. This is evident in nylon, which exhibited bleaching and surface deterioration, and in biodegradable polymers such as PBAT and PLA, which may have fragmented into sub-detectable particles. These findings highlight the need for further refinement of soil organic matter (SOM) removal protocols to preserve plastic integrity while allowing effective SOM elimination, which is crucial for the accurate detection of plastics using fluorescence microscopy.

While no significant differences in MP concentrations were detected between fluorescence microscopy and FTIR spectroscopy, fluorescence microscopy offers a more cost-effective and time-efficient approach compared to existing spectroscopic methods. Fluorescence microscopy requires just 15 min to scan a 47-mm diameter filter disk in both fluorescence and brightfield modes using a × 50 objective, enabling high sample throughput, which makes it particularly well-suited for environmental monitoring. In contrast, spectroscopic methods like FTIR and Raman micro-spectroscopy, despite technological advancements, remain slow and expensive. For example, Bergmann et al. [66] needed 4.5 h to analyse a small portion (14.1 mm × 14.1 mm) of a filter using µFT-IR imaging, making these methods impractical for high throughput environmental monitoring. In this study, an FTIR protocol was developed to analyse a full 25-mm diameter Anodisc filter in about 3–4 h; however, data processing and analysis for each sample still required a minimum of an additional 4 h.

Optical and fluorescence microscopy facilitate rapid analysis compared to FTIR or Raman (micro)-spectroscopy; however, these techniques do not enable chemical identification of MPs or provide detailed insights into their physicochemical properties, which can be achieved through FTIR or Raman spectroscopy [65]. While advanced FTIR methodologies, such as quantum cascade laser (QCL)-based systems, have been shown to enhance analytical efficiency [67, 68], the accessibility of such high-end instrumentation remains limited. Meanwhile, integrating fluorescence microscopy with spectroscopic techniques presents a complementary approach, enhancing both sample throughput and the accuracy of polymer identification. For example, Prata et al. [14] demonstrated the effectiveness of fluorescent NR tagging as a preparatory step for Raman spectroscopy, resulting in a more efficient and systematic workflow for comprehensive microplastic analysis.

Enhancing NR-stained fluorescence imaging for environmental monitoring still requires a number of important challenges to be addressed. These include minimising potential matrix interferences, understanding plastic properties and weathering, standardising staining procedures (NR concentration, staining duration, solvent, and heating conditions), and microscopic setup (excitation source, camera configuration, and image analysis parameters). Rigorous quality assurance and control are also essential for method harmonisation and standardisation, with criteria such as method recovery using universal MP standards, detection limits, and reproducibility needed to be incorporated. Implementing these recommendations will enhance research compatibility, advance fluorescence imaging, and improve the reliability of this method for monitoring environmental plastic pollution.

## Supplementary Information

Below is the link to the electronic supplementary material.

Supplementary file1 (DOCX 5655 KB)

## Data Availability

Data is available as follows: https://zenodo.org/records/14319057?preview=1&token=eyJhbGciOiJIUzUxMiJ9.eyJpZCI6ImEwY2FiMjkwLTIzMzItNDBhYy1hMjg2LWJiMDYwMzNkZjI0YiIsImRhdGEiOnt9LCJyYW5kb20iOiI5NzQ5ZGRhMjQ3ZGU0ZDNmMjM2YzY3ZGI4ZmM5MWE5NSJ9.cdQJy82afgYtInUwXI20dCBk4pMTKvoFkPokWaiocE9d4J4wYD86T53o2c-b48J8_c44V4n80nNe5QFp4QIt_A.
