# Supplementary material for: Towards quality-assured measurements of microplastics in soil using fluorescence microscopy
Source: Anal Bioanal Chem. 2025 Mar 10;417(11):2225–38. doi: 10.1007/s00216-025-05810-6 (PMC11996956; doi:10.1007/s00216-025-05810-6)
Supplement: Supplementary file 1 — Supplementary file1 (DOCX 5655 KB) [file 216_2025_5810_MOESM1_ESM.docx]

**Towards quality-assured measurements of microplastics in soil using fluorescence microscopy**

Nhu Phan^1^, Crispin Halsall^1^, Stoyana Peneva^2^, Olivia Wrigley^3^, Melanie Braun^3^, Wulf Amelung^3^, Lorna Ashton^4^, Ben W.J. Surridge^1^, John Quinton^1^

^1^Lancaster Environment Centre, Lancaster University, Lancaster, LA1 4YQ, UK

^2^ Wessling GmbH, Am Umweltpark 1, Bochum, 44793, Germany

^3^ Institute of Crop Science and Resource Conservation, University of Bonn, Bonn, 53115 Germany

^4^Department of Chemistry, Lancaster University, Lancaster, LA1 4YQ, UK

Email contact: [c.halsall@lancaster.ac.uk](mailto:c.halsall@lancaster.ac.uk), [q.phanle@lancaster.ac.uk](mailto:q.phanle@lancaster.ac.uk)

**SUPPORTING INFORMATION**

Number of pages:17

Number of tables: 4

Number of figures: 12

1. **Microplastic extraction from soil**

The extraction of microplastics from soil comprised of a density separation step and the removal of soil natural organic matter using a Fenton reagent. The microplastic content in non-spiked soil was screened using the same protocol (three background samples for three soil types). Microplastic content from spiked samples was subtracted with the number of particles found in the non-spiked soils to avoid false-positive results, as previous studies pointed out that natural organic matter causes false positives when staining with Nile red.

A density separation step was first applied to isolate MPs from soils, which exploits the buoyancy of MP particles in a higher-density solution of ZnCl_2_. The Sediment Microplastic Isolation (SMI) unit was utilized as a simple-to-use kit for density separation, with outstanding performance proven in previous studies. [45, 50] The SMI unit was assembled (with smaller dimensions, figure S4), cleaned, and purged before the introduction of 10 g of soil and 50 mL of ZnCl­_2_. After that, the ball valve was tightly locked, and the SMI was shaken vigorously under an orbital shaker for 2 hours to ensure full contact between the sample and ZnCl_2_ and to dissolve the gelatine. The valve was then set in the open position, and an additional 200 mL ZnCl_2_ was added, which was then allowed to settle overnight to allow dense particles to settle out. Once the ZnCl_2_ solution became apparent, the valve was carefully closed. The supernatant in the headspace was vacuum filtered through a stainless-steel mesh, retaining the zinc chloride for further recycling. The SMI headspace was rinsed thoroughly with HPLC-grade water to recover any remaining particles and remove ZnCl_2_. Stainless steel meshes were then transferred to a 500 mL beaker containing 20 mL of 0.05M FeSO_4_, followed by 10-minute sonication at room temperature to wash off any particles attached closely. The meshes were then rinsed with HPLC water, removed, and washed carefully for further use. To start the Fenton reaction, 20mL of H_2_O_2_ was added to the beaker. After 24h, the samples were filtered on glass fibre filters (GFFs) and submerged with 5-7 drops of 5 μm /mL Nile red solution using a glass pipette while filters were still laid on the filter head. After 10 min, filters were thoroughly rinsed with hexane and vacuum-filtered to discard any accumulated liquid. Finally, filters were carefully transferred onto and stored inside covered glass Petri dishes and left air-dried in the dark before being observed under a fluorescence microscope. Samples were analysed within one week after staining to avoid precipitation and quenching of Nile red.

1. **Fluorescence microscopy and automated digital image analysis**

Microscopic imaging was performed using a stereo zoom microscope (Zeiss Axio Zoom.V16) equipped with a long working distance high-aperture macro lens (Plan NeoFluar Z 1.0x/0.25, FWD 56mm) and a fast, sensitive 12Mpixel camera (Zeiss AxioCam 512mono). The microscope had an automated stage and image stitching function to capture the entire filter surface (Zen Blue 2.6 and Zen-pro software). Sample filters stained with Nile Red were directly illuminated with the 470 nm light (CoolLED) and observed through the green filter (GFP filter, emission 524/50 nm) in the darkroom. Green fluorescence was chosen over the red counterpart due to the better fluorescence of synthetic polymers, less fluorescent interference from natural organic matter, and lower background signal intensity in green compared to red fluorescence mode. [38, 51]. As almost no fluorescence signal was detected from black and dark-coloured microplastics, the bright field mode was added to quantify black microplastic particles, e.g., PBAT/PLA and potentially the brownish nylon fibres. Therefore, the whole filter images were obtained for both green fluorescence and bright field, all at a magnification of 50× and a sensitivity of 1 without pixel binning, using the stitching function and surface focusing with several local support points.

Automated particle recognition and quantification based on the fluorescent images were performed in Fiji-ImageJ. The stitched images of the whole filter area in the Carl Zeiss CZI Format (.czi) were converted into a TIFF file (Tag Image File Format) and processed according to the workflow displayed in Figure 2. Firstly, a smoothing operation approximating a Gaussian distribution (Gaussian blur filter, figure 2b) was applied. The original image was then subtracted with the Gaussian blurred image to reduce the particle detection runtime and prevent random noise from being falsely detected as particles (figure 2c). This step is beneficial when fluorescence background/noise is present, possibly due to the uncompleted wash of Nile red and the signal from strongly fluoresced materials. Gaussian blur windows (sigma/radius) were set between 100-500 pixels depending on the level of background interference. Afterwards, a global thresholding method was applied to segment the images into particles of interest and background, with colour and intensity parameters adjusted individually to each image to avoid selecting the background as much as possible. Normally, the grey value was set from 400-800 to 65535 (maximal grey value for the 16-bit images of 65535) for the detection of LDPE (dia. 20-150 μm) in the fluorescence mode and from 0 to 1200 (dia. 100-250 μm) for black PBAT/PLA in the bright field mode. The pixel intensity threshold was chosen based on experiment 2.5.1 so that the threshold value can pick as much of all the particles of interest. Fill Hole operation was then applied to compensate for particle penetration resulting from a large Gaussian window and global thresholding. After that, watershed segmentation was used to separate fluorescence particles that lay in proximity to each other and were agglomerated. Watershed segmentation, however, was not applied for bright-field images for the quantification of PBAT/PLA particles due to the potential overestimation of PBAT/PLA particles caused by cracking and the spike shape of cryomilled particles. Finally, particles were quantified based on Feret’s diameter, which is defined as the mean of all diameters over all angles. The lowest size limit of 20 μm was chosen due to the high uncertainty in quantifying particles smaller than that (result from experiment 2.5.1, figure 6) and the use of 6-μm stainless steel mesh during sample preparation. Larger size microplastics prepared from consumer materials (dia. 500-1000 μm) were counted manually instead of Image J due to a variety of shapes and fluorescence intensities induced by different polymers.

### Validation of the Fluorescent Staining Protocol with Environmental Samples

- 1. **Sampling soil**

Sampling soilThe study area includes Field 1 (54°00′52″ – 54°00′48″N, 002°46′47″ – 002°46′43″W) and Field 2 (54°00′52″ – 54°00′47″N, 002°46′34″ – 002°46′25″W), both located southeast of Lancaster, United Kingdom. The annual mean temperature is 10.5°C, with daily and annual precipitation recorded at 34.5 mm and 414.2 mm, respectively (Time and Date, 2023). Both fields serve as field stations for Lancaster University and have not been used for agriculture for decades. Additionally, the fields are enclosed by barriers to protect against potential contamination from animals and humans.

For each field, five sampling sites were randomly selected within a 4-meter margin from the field's edge. At each site, a 50 cm x 50 cm quadrat was used to define the sampling area. Soil was dug to a depth of 20-25 cm using a stainless-steel shovel to reach the ploughing depth, though deeper soil could not be obtained due to high soil compaction. The soil within the quadrat was thoroughly mixed to a 20 cm depth, and a subsample of 1-2 kg of wet soil was collected, stored in 100% cotton bags, and kept at 4°C until further analysis, including pH, conductivity, organic matter content, and particle size distribution.

- 1. **Microplastic extraction and Fourier transformed infrared microspectroscopy measurement**

For MP extraction, 50 g of each soil sample was dried at 40°C, homogenised and sieved through a 2 mm mesh. The sieved soil was then subjected to the previously described MP extraction process. However, after Fenton digestion, the extracted MPs were filtered through a stainless-steel filter. The particles deposited on the filter were sonicated, rinsed into a clean beaker, and transferred to a 100 mL volumetric flask with HPLC-grade water. From this, 15 mL subsamples were taken using a glass pipette under magnetic stirring for fluorescence microscope analysis. Additionally, 0.5–1.8 mL aliquots were used for FPA-µ-FTIR analysis.

For FTIR analysis, aliquots of samples were filtered on 25 mm Anodiscs™ (Whatman™, PP-supported, 0.2 µm pore size) and analysed via focal plane array micro-Fourier-transform infrared spectroscopy (FPA-µ-FTIR). A Bruker Hyperion 3000 FTIR microscope with a 64×64-pixel FPA detector and Bruker Tensor 27 FTIR spectrometer was used for imaging in transmission mode. Filters were placed on CaF_2_ windows (25 mm diameter, 2 mm thickness, Korth Kristalle, Germany). The entire surface of the filter was scanned using a 3.5× IR objective. Spectra were collected with a coaddition of 32 scans at an 8 cm⁻¹ resolution and a measuring range between 1250 and 3600 cm⁻¹. Pixel sizes of the measured data were about 11 µm. Imaging data were then compared against a reference database using siMPle (v. 1.0.1).

**Table S1:** Selected Physicochemical Properties of Agricultural Soils Used for Extractions

| Soil |  | Sand^(a)^ | Silt^(b)^ | Clay^(c)^ | organic-C (%) |
| --- | --- | --- | --- | --- | --- |
|  |  | Mass (%) | | |  |
| Sand |  | 73.3 | 16.5 | 8.5 | 0.9 |
| Clay |  | 9.9 | 36.3 | 50.3 | 5.9 |
| LUFA 2.4 |  | 22.4 | 42.2 | 23.7 | 1.83 |

1. Sand refers to soil particles with a diameter of 0.05-2.0 mm. ^(b)^ Silt refers to soil particles with diameters 0.002-0.05 mm. ^(c)^ Clay refers to soil particles with a diameter < 0.002 mm.

**Table S2**: Details of MPs used in the recovery experiments, including polymer type, form/shape, size, colour, and original product. Density data correspond to those of pure polymers. [23]

| Size range | Plastics | Form  shape | Colour | Source | Density  (g cm^-3^) |
| --- | --- | --- | --- | --- | --- |
| Consumer material plastics  500-1000 μm | LDPE | film | transparent | food packaging | 0.91–0.92 |
|  | PP | film | transparent | food packaging | 0.90–0.91 |
|  | PS | fragment | white | insulation board | 0.015-0.03 |
|  | Nylon | fibre | brown | carp fishing line | 1.13-1.41 |
|  | PET | fragment | transparent | food packaging | 1.37-1.45 |
|  | PBAT/PLA | film | black | agricultural mulching film | 1.23-1.29 |
|  | HDPE | fragment | white | milk bottle cap | 0.93-0.97 |
|  | PVC | fragment | transparent | insulated cable | 1.16-1.58 |
| 100-250 μm | PBAT/PLA | film | black | agricultural mulching film | 1.23-1.29 |
| ≤ 150 μm | LDPE | particle | white | Goonvean Fibre Ltd. | 0.91-0.92 |

**Table S3**: Characterization of soil samples collected from Hazelrigg field for the validation of the fluorescent staining methods.

| Sampling date | Field Area  (ha) | Sample code | Latitude | Longitude | Characterization at sampling point | | | Soil physiochemical characterization | | | | | |
| --- | --- | --- | --- | --- | --- | --- | --- | --- | --- | --- | --- | --- | --- |
|  |  |  |  |  | Moisture | Conductivity (dS/m) | Temperature | % C | pH | Conductivity (uS/cm) | Particle size analysis | | |
|  |  |  |  |  |  |  |  |  |  |  | % clay | % silt | % sand |
| 08/06/2023 | 0.59 | HF1-S1 | 54.01365 | -2.779300 | 16.4 | 0.20 | 21.0 | 14.2 | 5.9 | 166 | 28.5 | 45.2 | 26.3 |
|  |  | HF1-S2 | 54.01408 | -2.779208 | 15.4 | 0.20 | 22.9 | 12.8 | 5.7 | 191 | 19.4 | 27.5 | 53.1 |
|  |  | HF1-S5 | 54.01376 | -2.778979 | 30.9 | 1.50 | 24.7 | 13.3 | 5.5 | 222 | 21.7 | 33.3 | 45 |
|  |  | HF2-S3 | 54.01322 | -2.775445 | 9.1 | 0.10 | 30.4 | 10.6 | 5.1 | 117 | 20.9 | 40.7 | 38.4 |
|  |  | HF2-S4 | 54.01314 | -2.775675 | 10.6 | 0.20 | 31.0 | 10.7 | 5.2 | 137 | 17.4 | 32.5 | 50.1 |
|  |  | HF2-S5 | 54.01371 | -2.775629 | 7.0 | 0.00 | 28.7 | 10.6 | 5.2 | 125 | 15.8 | 27 | 57.2 |

**Table S4**: Number of SMP particles recovered from three soil types (including spiked soil, background and blanks).

|  | LUFA 2.4 | | Sandy | | Clayey | | Blank |
| --- | --- | --- | --- | --- | --- | --- | --- |
| Number of particles  ($\bar{\boldsymbol{X}}\boldsymbol{\pm SD}$) | spiked | background | Spiked | background | Spiked | background |  |
| LDPE  dia. 10-150 µm | 14010 $\pm$2130 | 2230 | 10830 $\pm$ 1780 | 540 | 5300 $\pm$ 1760 | 2620 | 130 |
| PBAT/PLA  dia. 100-250 µm | 4800 $\pm$480 | 82 | 1820 $\pm$ 820 | 222 | 715 $\pm$ 30 | 22 | 0 |
| Plastics  dia. 500-1000 µm | 39 $\pm$ 3 | 0 | 35 $\pm3$ | 4 | 38 $\pm$ 2 | 2 | 1 |

**Figure S1:** ATR-FTIR (a) and Raman (b) spectra of LMP prepared from consumer products using in the recovery experiment. All the spectra were normalised with the highest peak using Spectragryph software. Spectra were compared with OpenSpecy library and confirmed with their own labels.

|  | 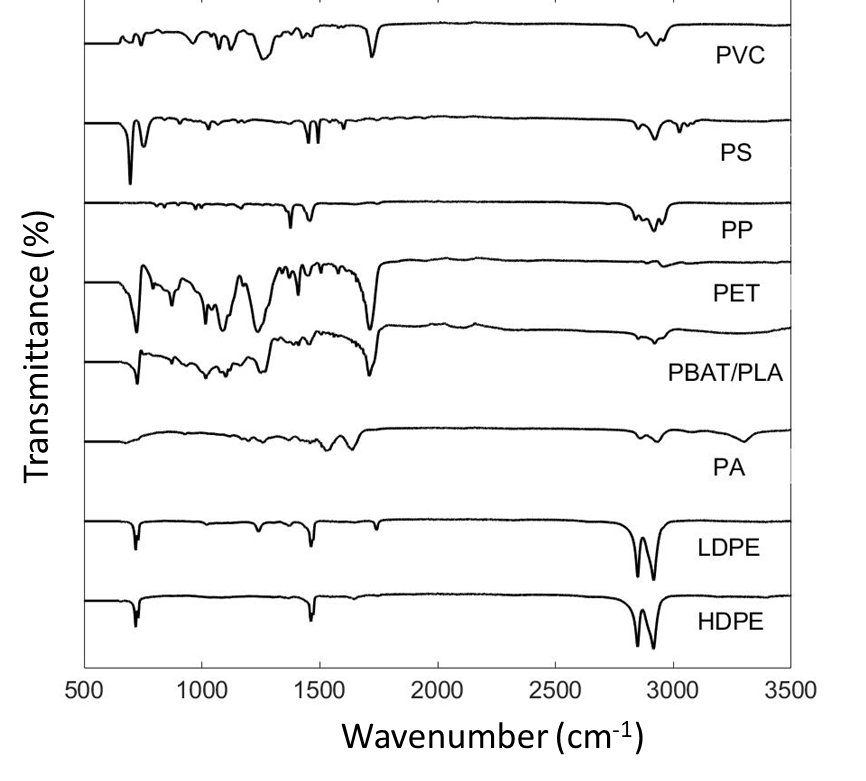 |  |  |
| --- | --- | --- | --- |
| b.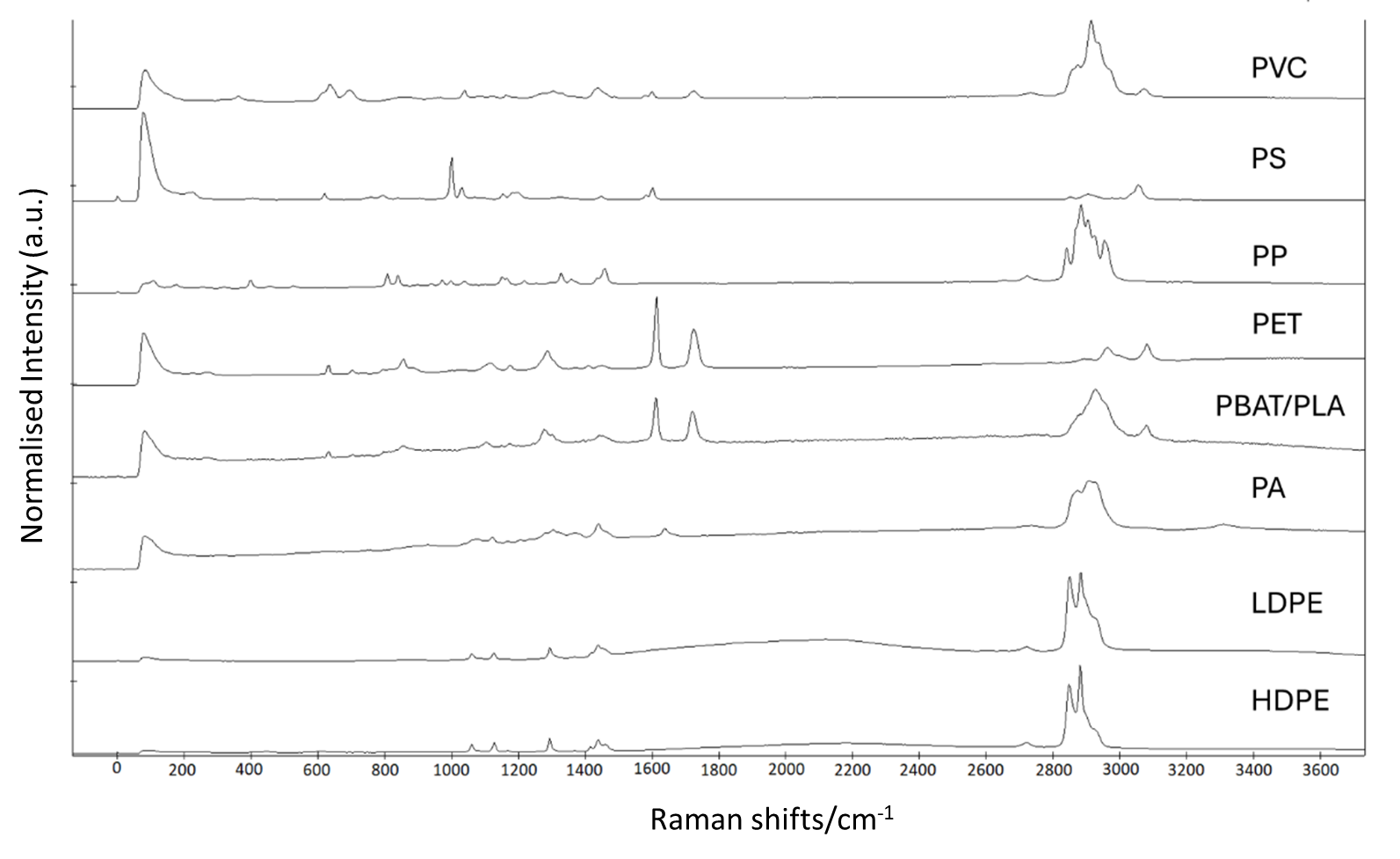 | | |  |

**Figure S2:** Microplastic spikes prepared in gelatin sheets: a) larger-sized microplastics (500-1000 μm) including eight type of plastics low-density and high-density polyethylene (LDPE and HDPE), polypropylene (PP), polystyrene (PS), polyethylene terephthalate (PET), poly (PA), polyvinyl chloride (PVC) and the biodegradable plastic polybutylene adipate-co-terephthalate/polylactide blend (PBAT/PLA). B) smaller-sized microplastics including black PBAT/PLA particles (dia. 100-250 μm) and white LDPE particles (dia. ≤ 150 μm)

| **a)** | **b)** |
| --- | --- |
| **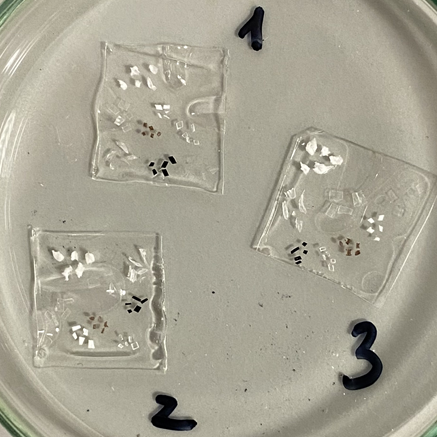** | **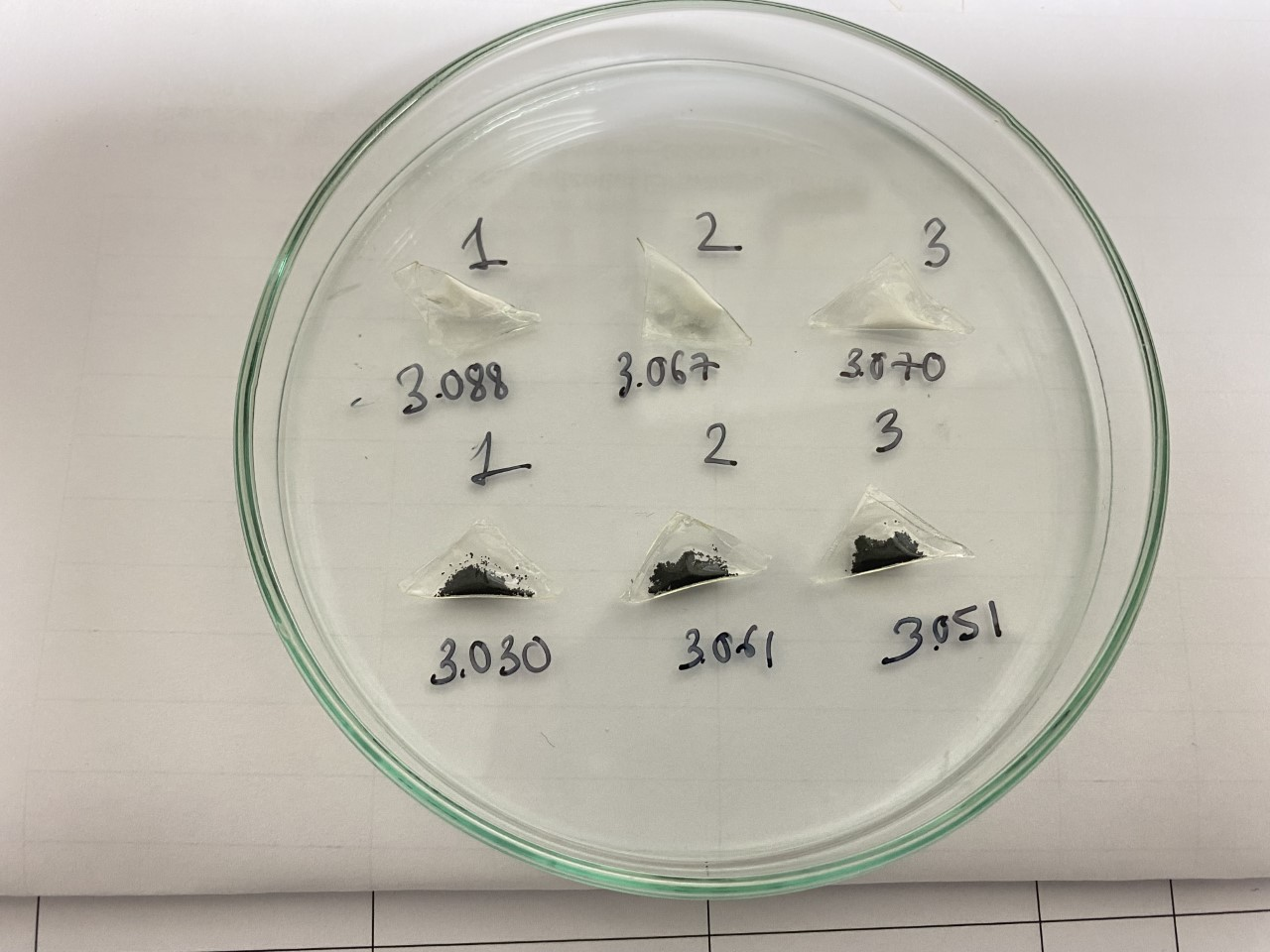** |

**Figure S3**. Microscopic images of the eight large MiPs used for the soil spiking. As it follows, A. polypropylene (PP); B. low density polyethylene (LDPE); C. high density polyethylene (HDPE); D. polystyrene (PS); E. polybutylene adipate terephthalate/polylactic acid (PBAT/PLA); F. polyamide – nylon 6,6 (PA); G. polyethylene terephthalate (PET) and H. polyvinyl chloride (PVC).

| **A.** | **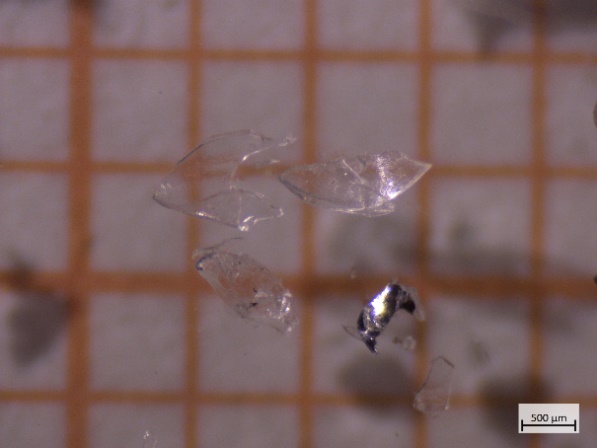** | **B.** | **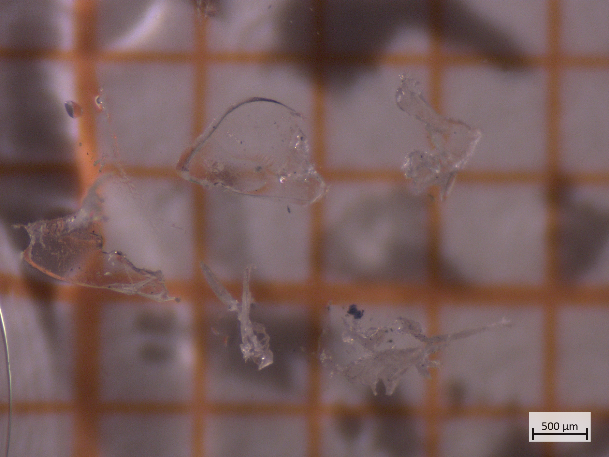** |
| --- | --- | --- | --- |
| **C.** | **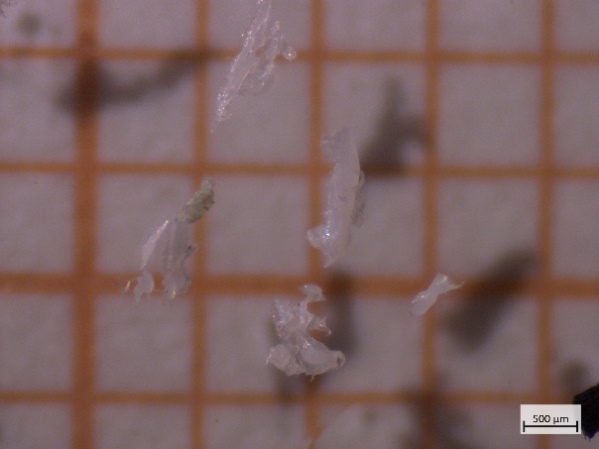** | **D.** | **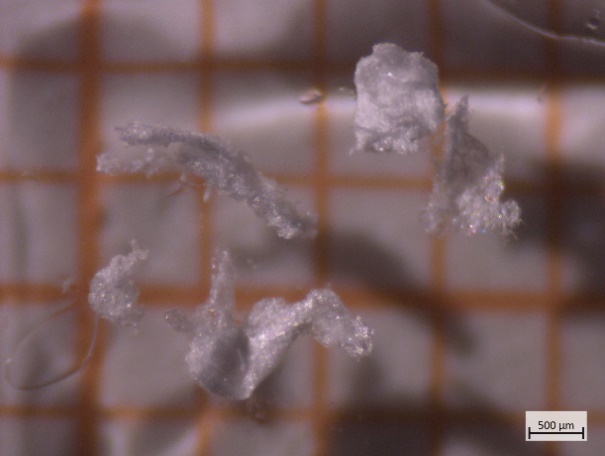** |
| **E.** | **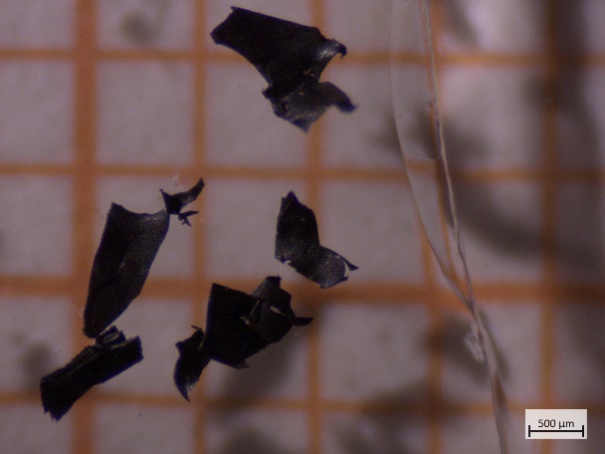** | **F.** | **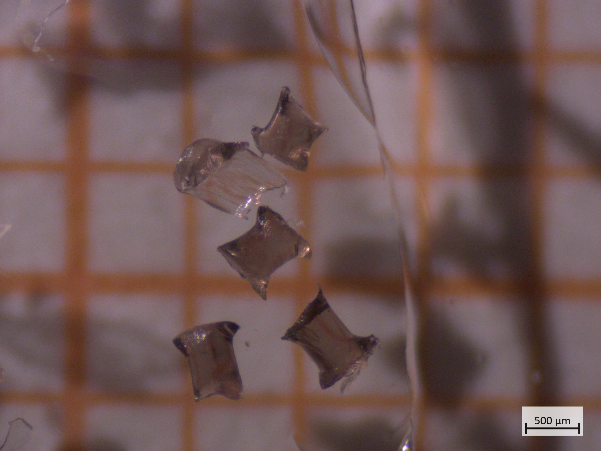** |
| **G.** | **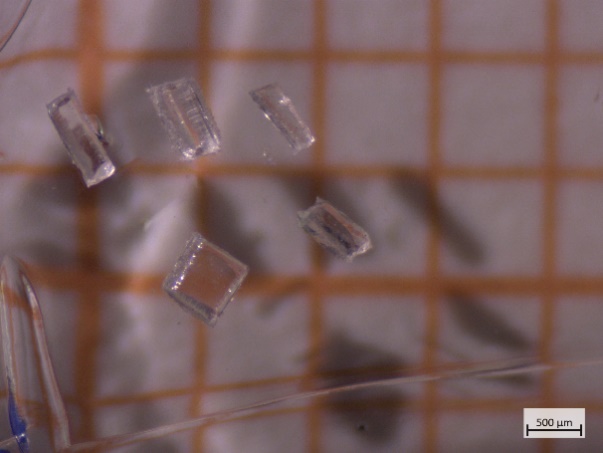** | **H.** | **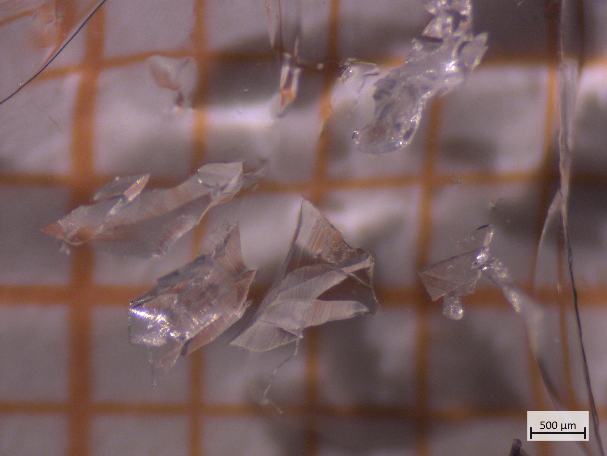** |

**Figure S4:** Dimensions for the Sediment Microplastic Isolation (SMI) unit using in density separation step.

| 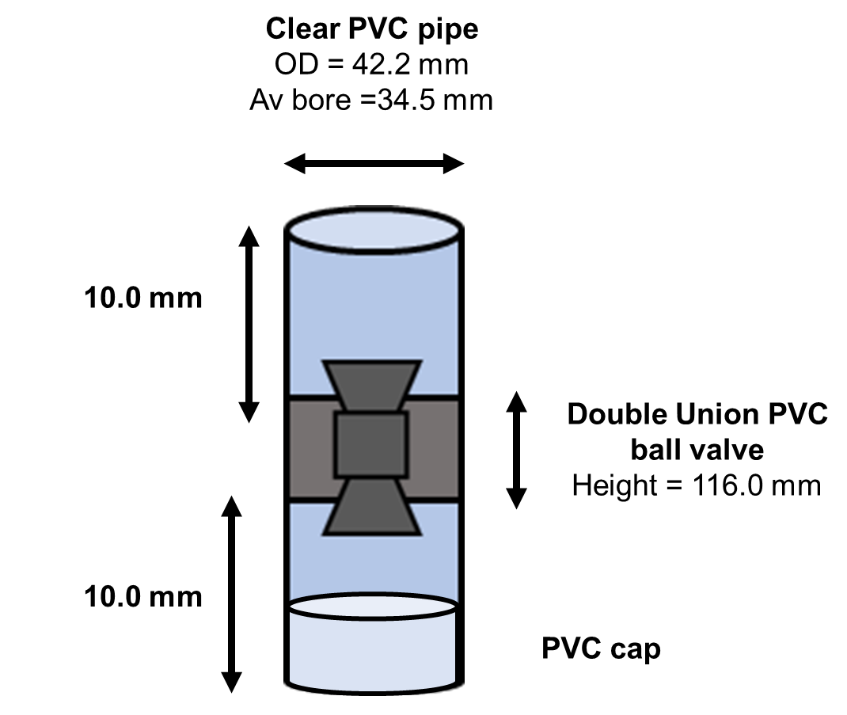 | 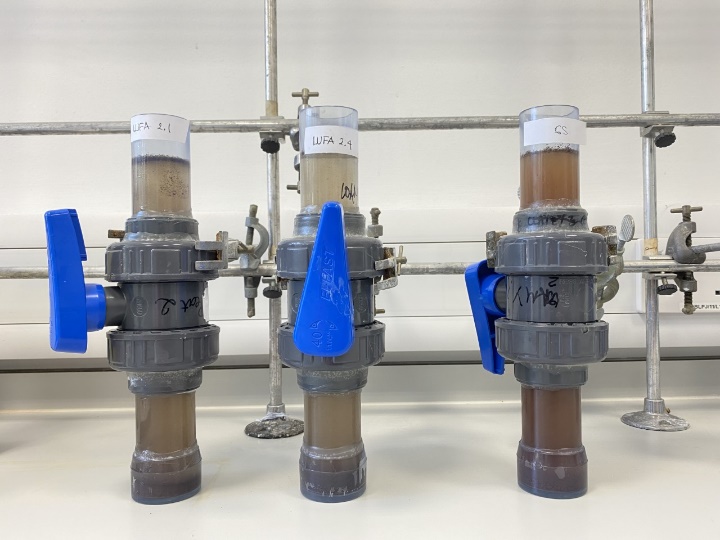 |
| --- | --- |
| **Figure S5**: Image processing scheme: a) cropped section of the original image; b) Gaussian blur image; c) Gaussian subtracted image; d) threshold image; e) filled hole image; f) Watershed segmentation image. | |

| 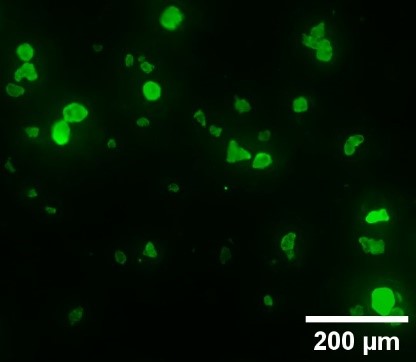  a) | 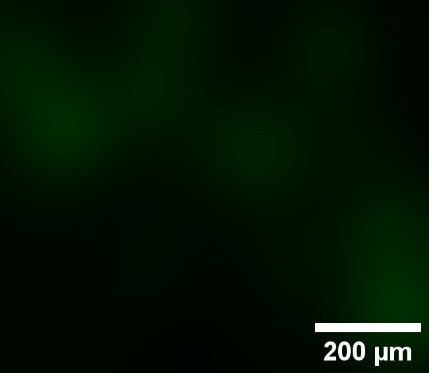  b) | 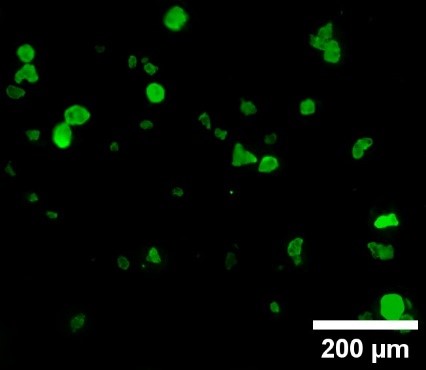  c) |
| --- | --- | --- |
| 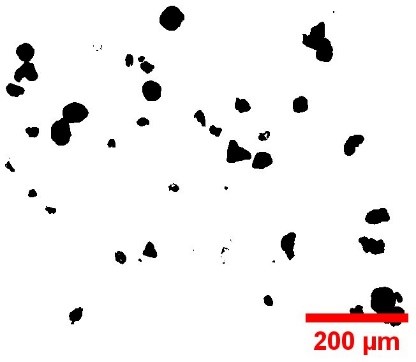d) | 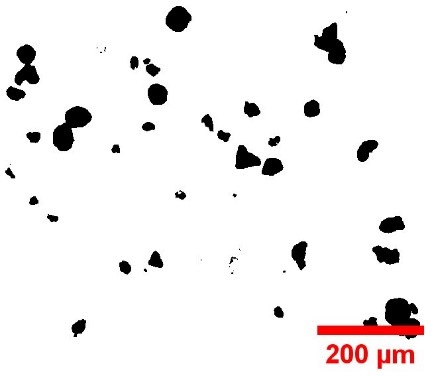e) | 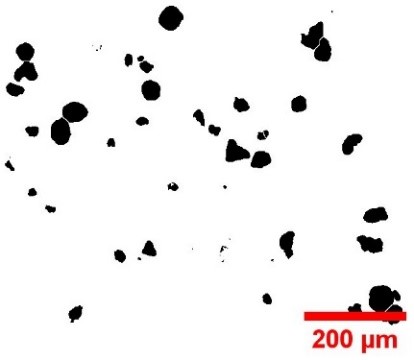f) |
|  |  |  |

| **Figure S6**: Bright-field (top) and Fluorescence images (bottom) of large MPs (dia. 500-1000 µm) prior to being spiked into soil. |
| --- |

| 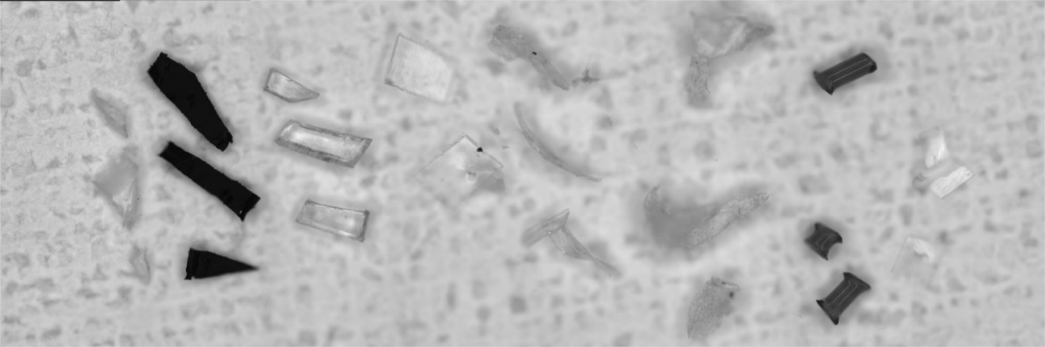 |
| --- |
| 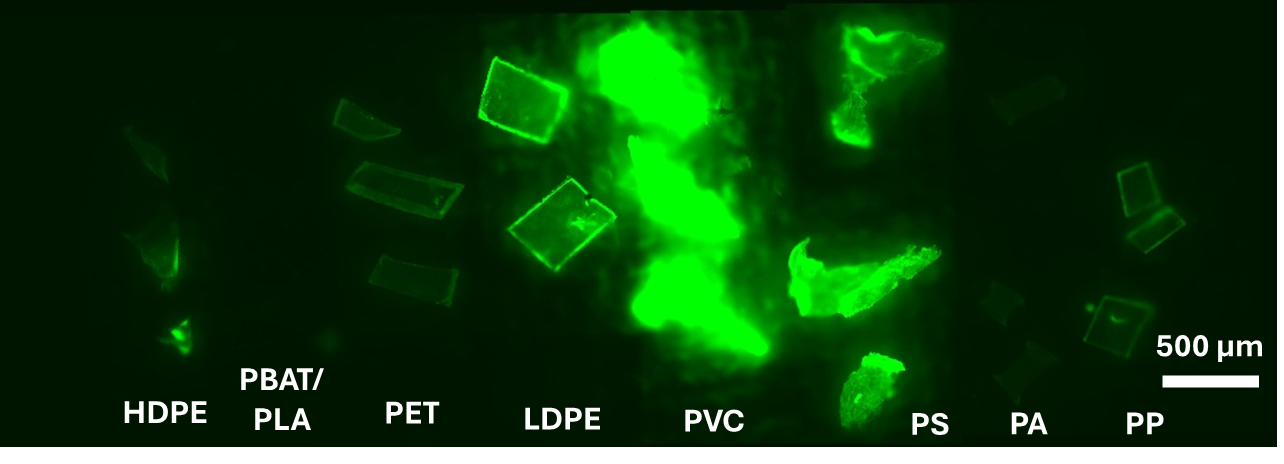 |

| **Fig. S7:** Boxplot showing the fluorescence intensity (a.u.) of microplastic particles stained with Nile Red across different polymer types, including HDPE, PBAT/PLA, PET, LDPE, PVC, PS, PA, and PP. Fluorescence intensity was calculated from six random points on each particle in the fluorescence images from Figure S6, covering both the edges and the particle center. The box represents the interquartile range (25%–75%), with the median indicated by the horizontal line. The whiskers extend to 1.5 times the interquartile range, and outliers are shown as diamonds. The mean fluorescence intensity is represented by square markers. |
| --- |
| 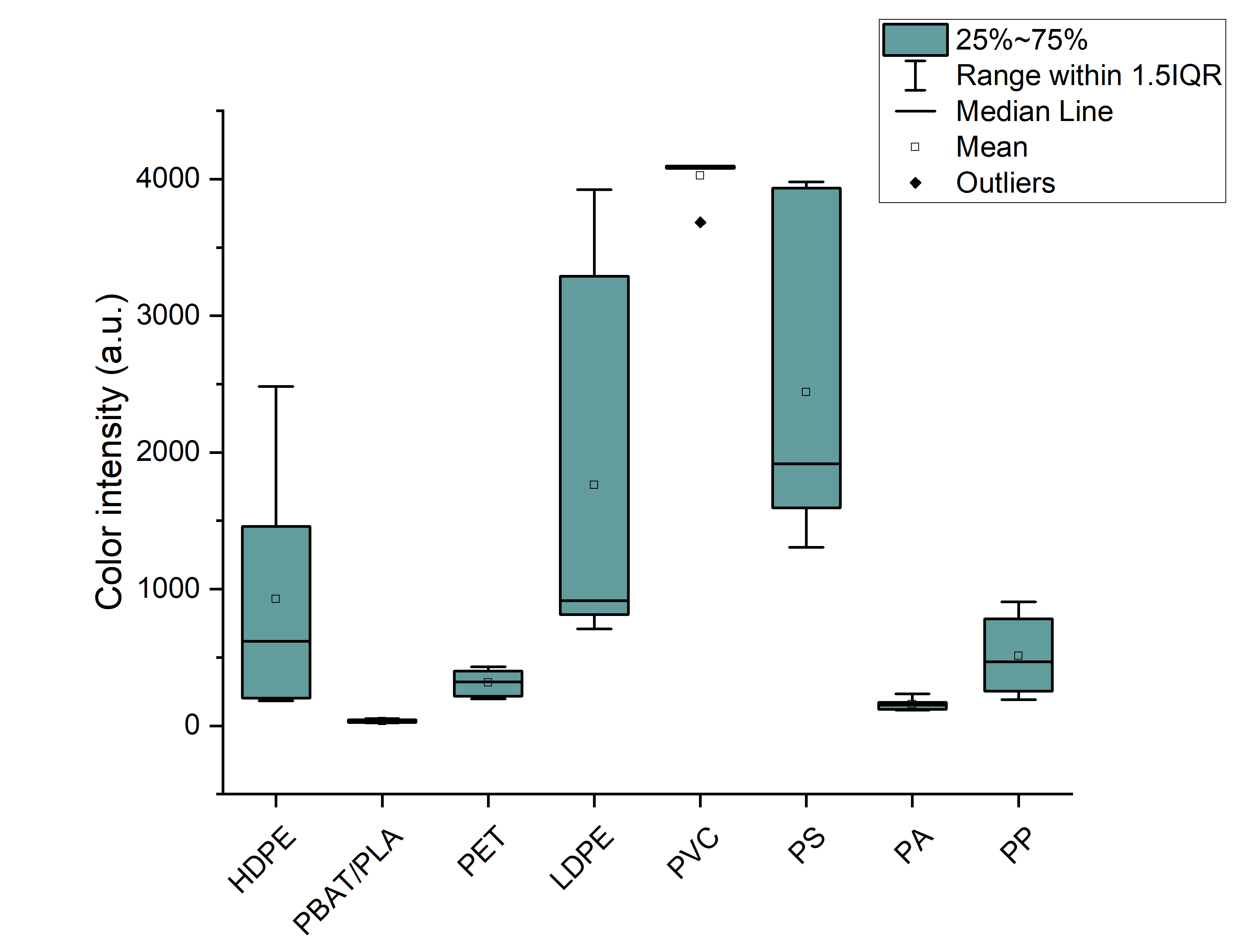 |
|  |

| **Figure S8**: Particle size distribution of PBAT/PLA SMPs (1) and LDPE SMPs (2) as assessed by optical and fluorescence microscopies coupled with ImageJ, respectively. The number of particles in each size category is reported as a mean (n=5) with their standard deviation. The light grey area corresponds to the sieving ranges used in the microplastic production method. | |
| --- | --- |
| 1) | 2) |

**Figure S9.** Particle size distribution of small MiPs from (1) LDPE and (2) PBAT/PLA obtained from particle counter analysis. Visualized on the x-axis - number of particles in 3 mg and on the y-axis – size of particles in µm. Average particle numbers found were 15733 +/- 138 particles in 3g for LDPE in a size range of 20-150 µm, and for PBAT/PLA we received 3200 +/- 30 particles in 3g in the size range of 100-250µm. These numbers served as a reference for the calculation of recovery rates.

| 1. |  |
| --- | --- |
| 2. |  |

|  |
| --- |
| **Figure S10:** Comparison of image analysis methods for fluorescent particle detection. The "MP-VAT" approach (left) shows challenges such as edge artifacts, halo effects caused by highly fluorescent particles, and difficulty in resolving clumped particles. The Fluorescence Image (center) illustrates the raw fluorescence image with background interference. The proposed approach in this study (right) demonstrates improved detection and segmentation of particles, effectively addressing edge artifacts, mitigating halo effects, and resolving clumped particles for enhanced accuracy. |
| 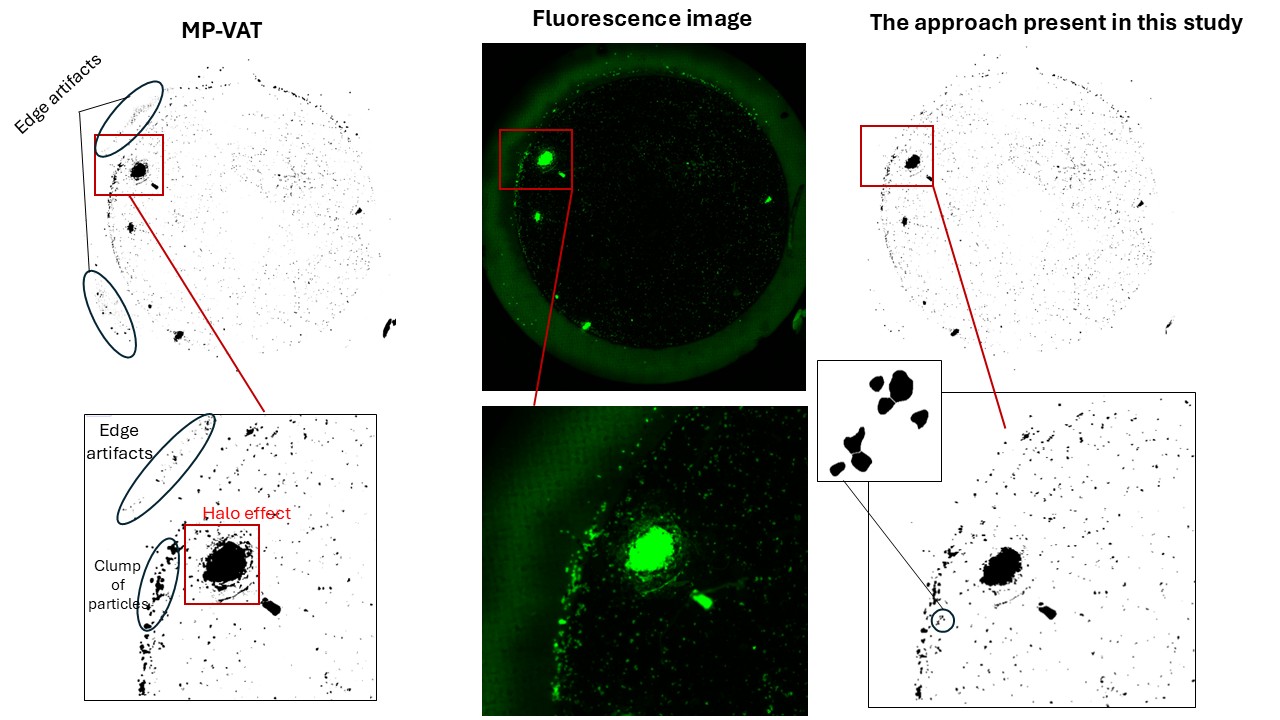 |
|  |

**Figure S11** Images of filters Comparison images of fluorescence images A: procedural lab blank, B: non-spiked background soil, and C: Plastic-spiked soil sample and bright-field images: C: procedural lab blank, D: non-spiked background soil, and E: Plastic-spiked soil sample and bright-field images. All samples were treated using the same workflow described previously.

| 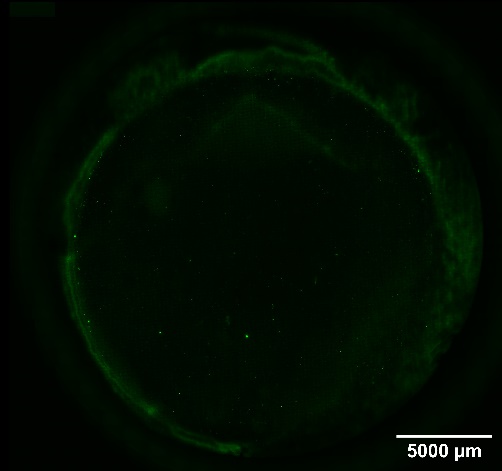 | 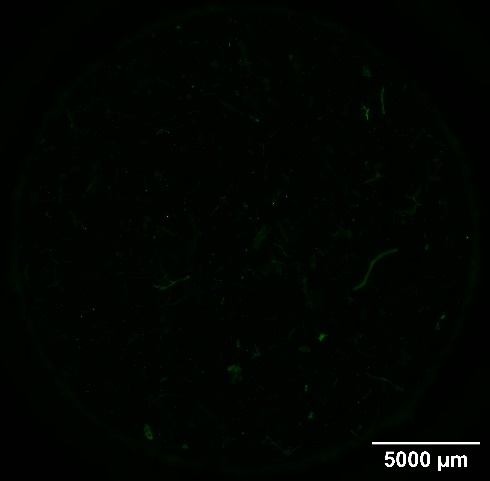  b) | 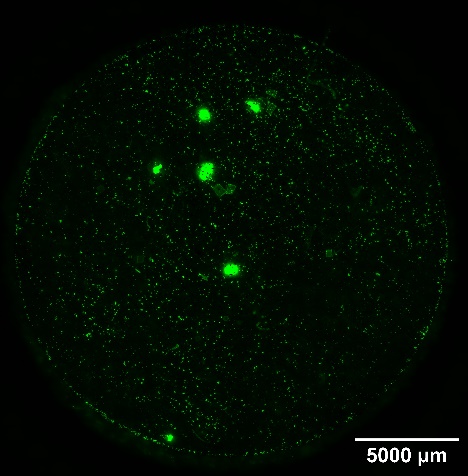  c) |
| --- | --- | --- |
| 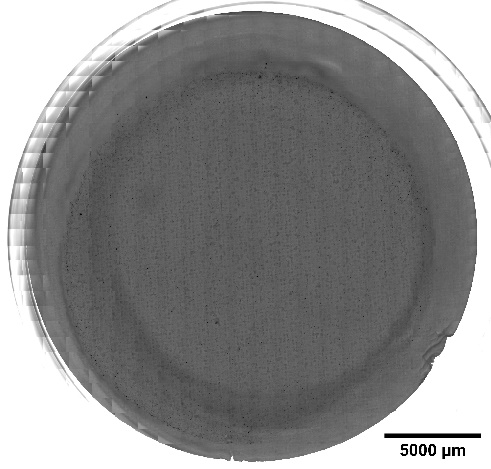  d) | 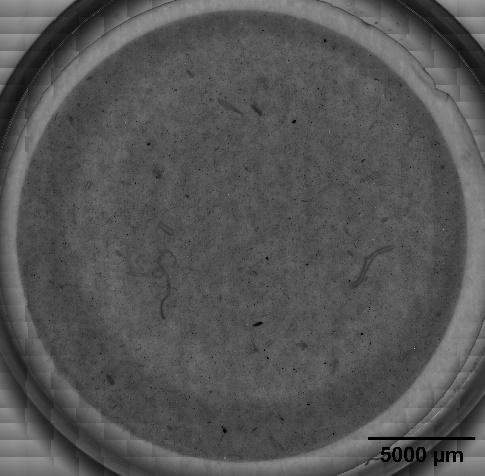  e) | 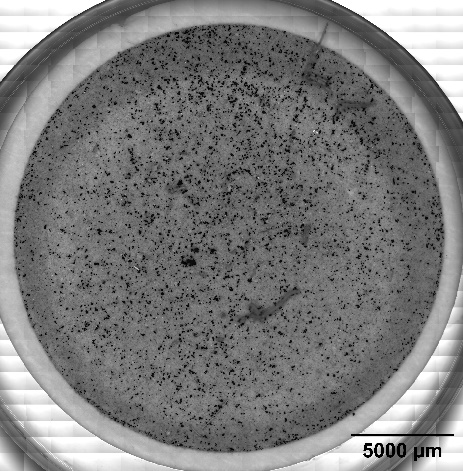 |

**Figure S12**: Correlation between fluorescence particles (a) and black particles (b) with natural organic matter content for non-spiked background soils. Organic matter content was 5.90%, 1.83% and 0.83%, respectively, for clay, LUFA 2.4 and sandy soils.

| a) | b) |
| --- | --- |
|  |  |

**Figure S13**: Size distribution of fluorescence particles detected soil backgrounds.


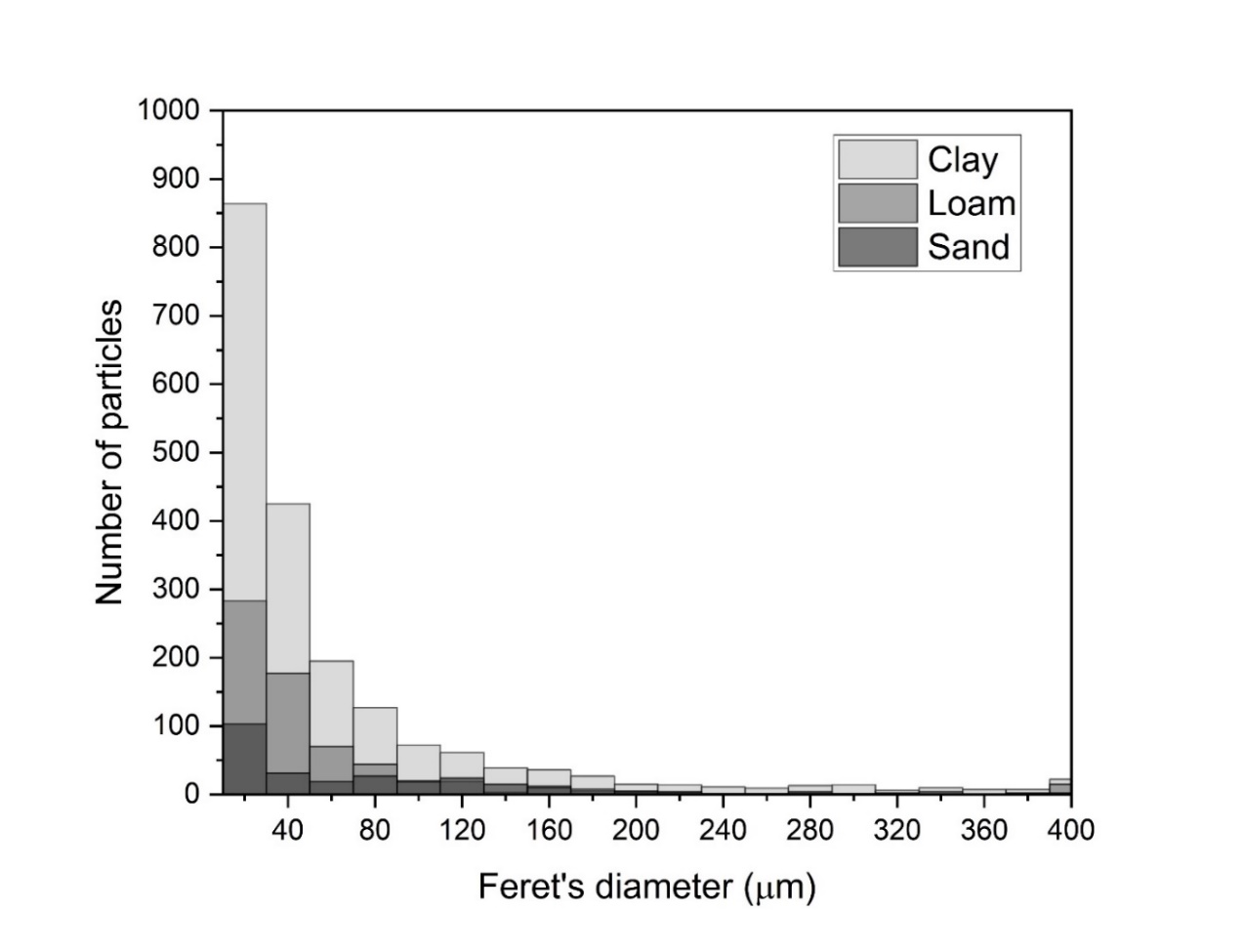


**Figure S14:** Microplastic number concentrations of soil samples collected from the Hazelrigg field station measured by Nile-red staining- fluorescence microscopy and by FTIR micro-spectroscopy method.
